# Controlling dodder (*Cuscuta planiflora*) in Egyptian clover with silica nanoparticles and a novel bioherbicide

**DOI:** 10.1038/s41598-025-16004-6

**Published:** 2025-08-25

**Authors:** Hend Mohammad Saad Ibrahim, Ibrahim E. Soliman, Mohamed E. Z. Kenapar, Sally F. Desoukey, Yasser M. Shabana, Abd ElAziz T. Bondok

**Affiliations:** 1 Agricultural Botany Department, Faculty of Agriculture, Cairo University, Giza, 12613 Egypt; 2 Weed Research Central Laboratory, Agriculture Research Center, Giza, 12619 Egypt; 3 Plant Pathology Department, Faculty of Agriculture, Mansoura University, El-Mansoura, 35516 Egypt; 4 National Council of Agricultural and Food Research, Academy of Scientific Research and Technology, Cairo, 11516 Egypt; 5 Forage Crops Research Department, Field Crops Research Institute, Agriculture Research Center, Giza, 12619 Egypt

**Keywords:** Egyptian clover, *Cuscuta planiflora*, Bioherbicides, Si nanoparticles, Glyphosate, *Fusarium incarnatum*, Biotic, Parasitism

## Abstract

Dodder (*Cuscuta planiflora*) is a major parasitic plant species affecting the productivity of Egyptian clover (*Trifolium alexandrinum* L.), an important forage crop in Egypt. A field trial was executed on Egyptian clover heavily infested with dodder during the winter seasons of 2021/2022 and 2022/2023. The aim of this study was to investigate the effect of cultivar choice and seven dodder control treatments on dodder growth performance as well as growth and yield components of Egyptian clover under infestation conditions. Results indicated that Helali was the most tolerant cultivar to dodder infestation. Moreover, treatments using *Fusarium incarnatum*-based bioherbicide, silica nano-particles (Si-NPs), and chemical herbicide (glyphosate) effectively controlled dodder after parasitism. The application of Si-NPs at 22 and 30 g fed^−1^ and bioherbicide at 20 and 30 kg fed^-1^ effectively reduced dodder biomass while increased biomass and seed yield of infested clover. The interaction between the Helali cultivar and Si-NPs at 30 g fed^−1^ was the most effective in reducing dodder biomass. Anatomical investigation of the stem revealed that Helali exhibited the greatest tolerance against dodder penetration. The electrophoretic protein profile indicated an unchanged protein pattern for Helali under infestation conditions. These findings suggest that Helali possesses a robust defense system and genetic diversity, making it the most tolerant cultivar to dodder infestation in conjunction with the high efficacy of the dodder control treatments used in this study.

**Supplementary Information:**

The online version contains supplementary material available at 10.1038/s41598-025-16004-6.

## Introduction

Dodder (*Cuscuta planiflora* Ten.) is considered the most threatening weed species that negatively affects the productivity of Egyptian clover (*Trifolium alexandrinum* L.) both quantitatively and qualitatively in Egypt^1^. Egyptian clover, commonly known as berseem, is a winter annual harvest crop widely cultivated in Egypt and several temperate and subtropical regions for fresh fodder, silage and hay, while also enhancing soil fertility as a major leguminous crop^2^. The productivity and quality of such an important fodder crop are significantly compromised by the use of uncertified local seeds that may be contaminated with weed seeds, such as dodder (*Cuscuta* spp.)^3^. *Cuscuta* spp. are among the most damaging and widespread stem-holoparasitic plants, lacking photosynthetic capability^4,5^. *Cuscuta* spp*.* depend on host plants to complete their life cycle via absorption of nutrients and necessary elements from the host by a specialized organ called haustorium^6^. Several studies have documented the harmful effects of dodder on Egyptian clover. For example, Al Shair^7^ reported that dodder decreased the biomass of the first and second cuttings of Egyptian clover in addition to declination of final seed yield. Similar reductions in fresh weight and seed yield due to dodder infestation were reported by Soliman and Abd El-Hamid^8^. Moreover, Leilah et al.^9^ found that *C. planiflora* decreased fresh, dry, and seed yields of infested Egyptian clover in two consecutive seasons.

Selection of resistant or tolerant genotypes is an effective strategy for mitigating the harmful effects of parasitic weeds such as dodder^5^. For example, “Helali” genotype was reported among the most tolerant genotypes of Egyptian clover to dodder infestation^3,10^. El-Refaey et al.^11^ attributed such tolerance of the Helali genotype to its relatively higher content of phenolic acids in leaves. Moreover, Egyptian clover cultivars such as Gemmiza 1, Giza 6, Helali, and Sakha 4 exhibited lower dodder infestation rates associated with increments in forage biomass and a higher tolerance index^1^.

Glyphosate (N-phosphonomethyl-glycine) is the most commonly used herbicide with high efficacy for weed control, especially dodder, in Egypt^5,8,12,13^. Nevertheless, many weeds have evolved resistance against it, in addition to many environmental and human health concerns related to its potential toxicity and carcinogenicity^14^.

Recently, the use of nanoparticles has emerged as a safer, more cost- and resource-effective alternative to chemical herbicides. For instance, Silica nanoparticles (Si-NPs) are extensively used in agriculture as pesticides, fungicides and herbicides^15,16^. Si-NPs are characterized by having small particle size with high surface area relative to their volume, which improves their penetrability to plant and/or pathogen tissues and increase their biochemical reactivity^16,17^. These characteristics allowed Si-NPs to be nano-carriers capable of transferring different materials including pesticides and herbicides into plant tissues with high efficiency^16^. Moreover, Si-NPs are environmentally friendly owing to their ability to re-enter ecosystems^16–18^. Si-NPs have a vital role in alleviation of biotic stress induced by pathogens such as bacteria, fungi and parasitic weeds^16–19^. The existing literature focused on the mechanisms by which Si- NPs effectively mitigates plant pathogenic attacks^16–19^. However, its practical efficiency in control of plant parasitic species such as *Cuscuta* remains unexplored. Accordingly, few reports were available on the role of Si-NPs in the control of dodder^20,21^.

Alternative weed control strategies involve the use of natural antagonists, known as “biological control”^5,22^. In the recent decades, *Fusarium* spp., soil-borne fungi, aroused as potential bioherbicides for several weed species^23^. Among these, *Fusarium oxysporum*, *F. solani*, and *F. incarnatum* are dominant species used for control of parasitic plants such as *Orobanche*, *Striga* and *Cuscuta* spp.^22–26^. They are characterized by being host-specific, capable of affecting the target weed species without harming the host plant, in addition to its ability to suppress weed growth during pre-emergence stages due to their long persistence in soil^22,27^. *F. incarnatum*, in particular, was repeatedly reported for controlling *Orobanche*^22–24^. However, its potential role in controlling dodder is not investigated yet.

The present investigation was conducted to study the effect of dodder infestation on Egyptian clover production and to explore the use of *F. incarnatum*-based granular bioherbicide and Si-NPs as alternatives to the chemical herbicide glyphosate for controlling dodder during its early developmental stages, ultimately enhancing clover production under infestation conditions.

## Materials and methods

### Experimental procedure and plant material

A field trial was conducted during two successive winter seasons of 2021/2022 and 2022/2023, at the Gemmiza Agricultural Research Station, Gharbia governorate, Egypt (30° 797′ N latitude, 31° 124′ E longitude). Mechanical and chemical analyses of experimental soil are presented in Table 1, following the methods outlined by Jackson^28^. Average weather data during the two growing seasons are presented in Table 2.

**Table 1:** Mechanical and chemical properties of the experimental soil (0–30 cm depth) in 2021/22 and 2022/23 seasons.

Seasons	Particle size distribution			Soil texture	Chemical analyses					
	Sand %	Silt %	Clay %		EC (ds m^−1^) (1:5)	pH (1:1)	Organic matter%	Available (mg kg^−1^)		
								Total N (%)	P (ppm)	K (ppm)
2021/2022	19.3	31.4	50.3	Clay	2.21	7.76	1.40	29.0	6.03	412.1
2022/2023	18.1	29.2	48.7	Clay	2.75	7.80	1.51	31.2	7.01	376.3

**Table 2:** Weather data at Gemmiza Agricultural Research Station, Gharbia governorate, Egypt.

Month	Temperature (°C)		RH (%)	Rainfall (mm)
	T_max_	T_min_		
October	34.30	20.11	53.48	0.90
November	25.65	15.25	61.09	17.22
December	23.53	11.51	60.34	1.13
January	22.16	9.64	61.74	8.09
February	22.35	9.48	62.68	33.10
March	23.68	10.15	16.91	59.88
April	31.62	12.54	21.9	40.44

The data presented are average monthly means for the seasons 2021/2022 and 2022/2023.

T_max_: Average maximum air temperature, T_min_: average minimum air temperature, RH: average relative humidity, Rainfall: average precipitation.

Source of weather data: Egyptian Ministry of Agriculture and Land Reclamation, Agricultural Research Center, Central Lab. for Agricultural Climate.

The seeds of three Egyptian clover cultivars (Gemmiza 1, Giza 6 and Helali) were obtained from the Forage Crops Research Department, Field Crops Research Institute (FCRI), Agricultural Research Center (ARC), Giza, Egypt, and were sown on 20 and 24 October in the two seasons, respectively. The seeding rate was 20 kg fed^−1^, drilled in rows. NPK fertilization was applied according to the recommended doses. Dodder seeds were mixed with soil at 5% of clover seeds (w/w), following a preceding summer crop of *Zea mays* L. in both seasons. Irrigation was practiced monthly by surface flooding, totalling six irrigations per season.

Four cuttings were taken for each cultivar during the two growing seasons. The first cutting was taken 50 days after sowing (DAS). Subsequent cuttings were performed at monthly intervals after the first (80, 110, and 140 DAS). Following the fourth cutting, clover cultivars were allowed to grow for seed production and the clover seeds were harvested on 30 June 2022 in the first season and 25 June 2023 in the second.

### Experimental design and treatments

**Fig. 1:**
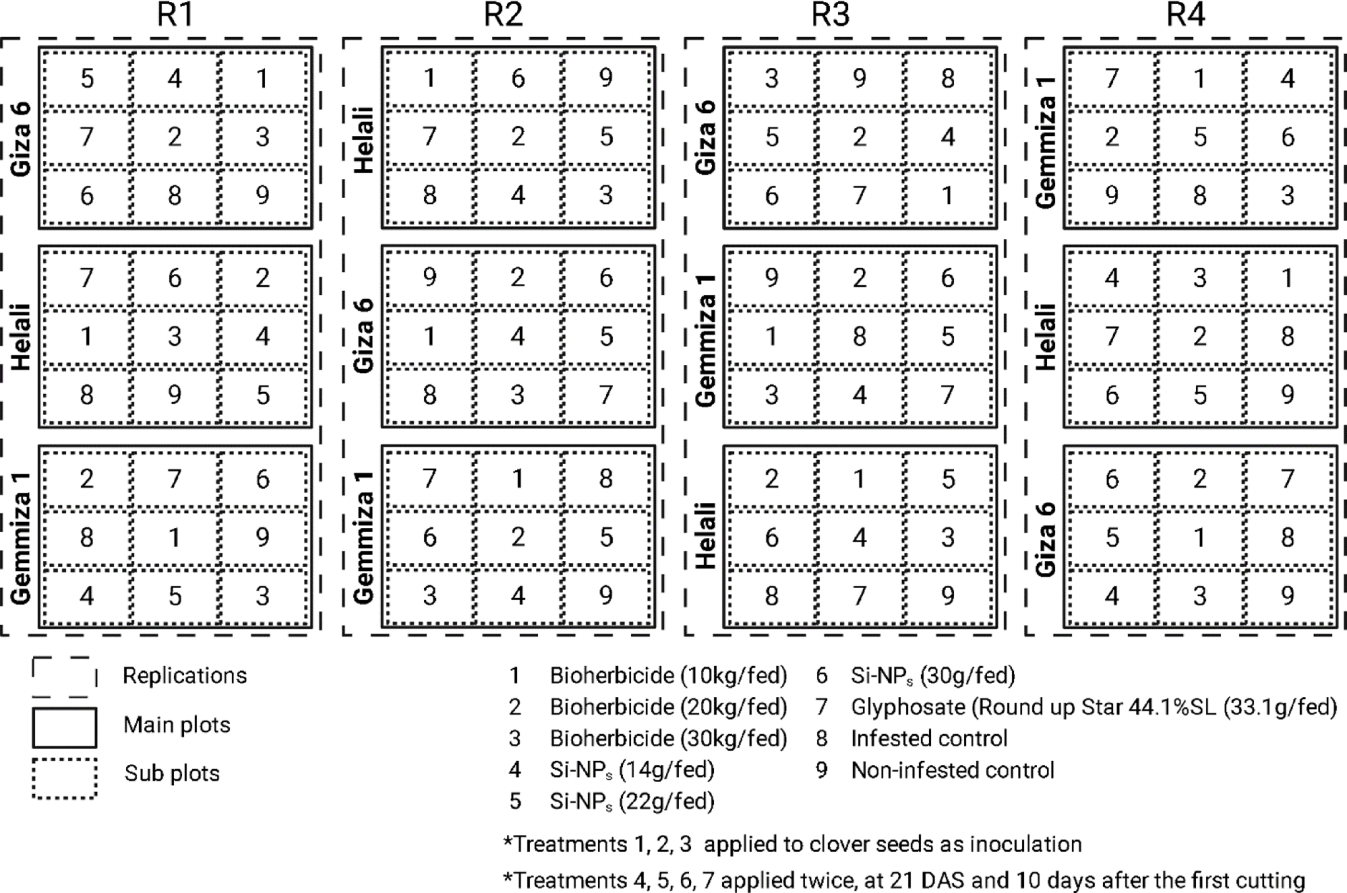
Experimental design and assignment of treatments (designed using BioRender).

A split-plot design with four replications was used for the experiment (Fig. 1). The main plots were assigned to the Egyptian clover cultivars. Meanwhile, the control methods for dodder were randomly distributed at the sub plots. Each plot area was 10.5 m^2^.

A. The main plots; Egyptian clover cultivars:
  1. Gemmiza 1.
  2. Helali.
  3. Giza 6.
B. Sub-plots; Dodder control methods:
  1. Bioherbicide at 10 kg fed^−1^, inoculated with clover seeds.
  2. Bioherbicide at 20 kg fed^−1^, inoculated with clover seeds.
  3. Bioherbicide at 30 kg fed^−1^, inoculated with clover seeds.
  4. Si-NPs at 14 g fed^−1^ applied twice, at 21 DAS and 10 days after the first cutting.
  5. Si-NPs at 22 g fed^−1^ applied twice, at 21 DAS and 10 days after the first cutting.
  6. Si-NPs at 30 g fed^−1^ applied twice, at 21 DAS and 10 days after the first cutting.
  7. Glyphosate (Round up Star 44.1% SL) at 33.1 g (a.i.) fed^−1^ applied twice, at 21 DAS and 10 days after the first cutting.
  8. Infested control.
  9. Non-infested control

### Bioherbicide source and description

A granular bioherbicide formulated from an isolate of *Fusarium incarnatum* (Desm.) Sacc. (isolate #2) (AUMC No. 9458) was obtained from the Plant Pathology Department at the Faculty of Agriculture, Mansoura University, Egypt.

### Source of Si-NPs

**Fig. 2:**
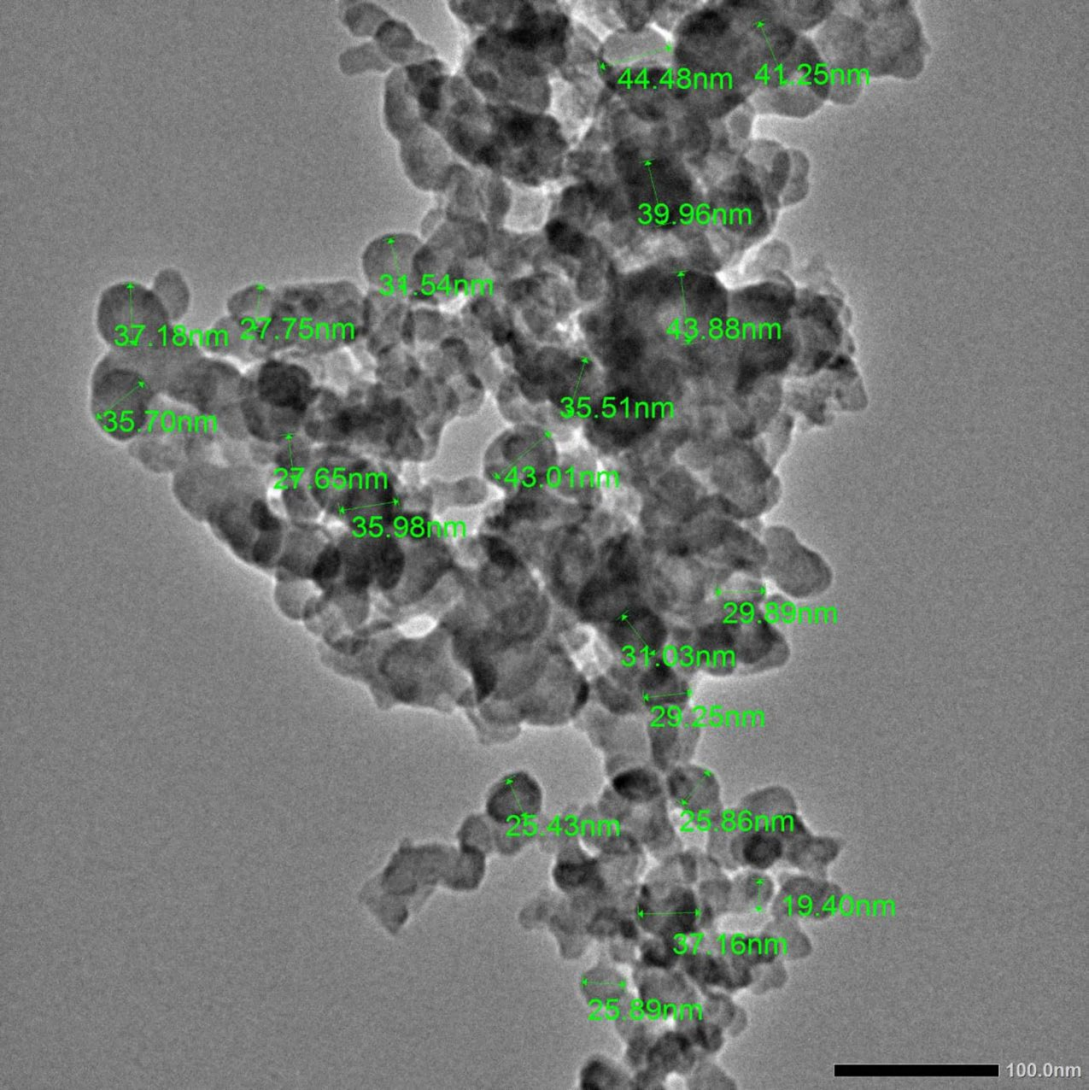
Si-NPs as captured by transmission electron microscope (TEM). Scale bar = 100 nm.

Si-NPs were procured from Nano Fab Company, Giza, Egypt, where they were prepared mechanically by ball milling, which is a top-down technique for preparation of nano-particles^29^. The purchased nano-particles were examined by JEOL (JEM-2100 PLUS) transmission electron microscope (TEM) at 200 kV to check for average particle size. The Si-NPs ranged in size from 19.40 to 44.48 nm (Fig. 2).

### Detection of genotoxicity of Si-NPs on Egyptian clover

DNA was extracted from fresh leaves of Helali cultivar. The DNA extraction process involved using the GeneJET genomic DNA purification kit protocol (K0721, Thermo Fisher Scientific Inc., Waltham, MA, USA), comprising the steps of lysing cells with lysis solution and proteinase K, ethanol precipitation, and elution. The PCR master mixture preparation was conducted under biosafety conditions, utilizing the Ready-To- Go RAPD analysis kit with the investigated primers. Agarose gel electrophoresis was employed to visualize and detect the amplification products. A 1.5% agarose solution was prepared, poured into a gel bed, and allowed to solidify. The gel was subjected to electrophoresis at 80 V for 100 min. After electrophoresis, the gel was stained with ethidium bromide for 30 min. and de-stained in distilled water for 20 min.

The assessment of genotoxicity of the three used Si-NPs doses (14, 22, and 30 g fed^−1^) was performed through the assessment of genomic template stability (GTS) in Egyptian clover via Random Amplification of Polymorphic DNA (RAPD) fingerprinting technique. GTS was quantified for each primer using the equation of Salarizadeh and Kavousi^30^.

$${\text{GTS}}\left( \% \right) = \left( {1 - \frac{a}{n}} \right) \times 100$$

where “a” is the average number of polymorphic bands in treated groups and “n” is the total number of bands in control samples. Polymorphic bands in the Random Amplified Polymorphic DNA (RAPD) analysis indicated variations compared to the control profile. The amplified bands were documented as 0 for absence and 1 for presence using Totallab software analysis (www.totalalb.com).

### Recorded agronomic traits

#### Dodder growth parameters

Dodder was sampled using a quadrant frame measuring 0.25 m^2^ (50 × 50 cm). Sampling was performed four times randomly per each sub-plot. Fresh and dry weights (g m^−2^) of dodder were recorded 45 DAS for the first cutting, 75, 105, and 135 DAS for the second, third, and fourth cuttings, respectively, in both growing seasons.

#### Clover growth parameters

Fresh and dry weights (ton fed^−1^) of clover were measured for each cutting in the two respective seasons, while number of seeds/ head, 1000 seed weight (g), and seed yield (kg fed^−1^) were measured at the end of both growing seasons.

### Statistical analysis

The obtained data for dodder and clover agronomic traits during each growing season were subjected to proper statistical analysis of variance (ANOVA) according to Snedecor and Cochran^31^. Following ANOVA, the least significant differences (LSD) at a 5% level of significance were calculated to compare the means^32^. Statistical analysis was performed using MSTAT-C software. Data were presented in tables as mean values.

### Clover stem microscopy

Stem specimens were cut throughout the basal internodes of the Helali, Giza 6 and Gemmiza 1 cultivars. The samples included healthy plants as control, dodder-infested plants, and dodder-infested plants treated with the granular herbicide (20 kg fed^−1^), resulting in a total of nine samples. Sample preparation was done according to Nassar and El-Sahhar^33^ as follows: stem specimens were fixed in formaldehyde aceto-alcohol (FAA) solution, dehydrated in tertiary-butyl alcohol series, and embedded in paraffin wax. Paraffin blocks were then sliced to a thickness of 20 µm using a rotary microtome. The last step was double staining of slides using crystal violet-erythrosine stains, and then mounting using Canada balsam. Stem transverse sections were photomicrographed at Cairo University Research Park (CURP), Giza, Egypt, using a Leica light image analysis system DM 750. Measurements in micrometers were taken for some stem histological parameters to assess changes that occurred in response to infestation with *Cuscuta* spp. and treatment with the bioherbicide.

### Protein electrophoresis (SDS-PAGE) procedure

Plant leaves (10 g), taken from healthy plants, dodder-infested plants, and dodder infested plants treated with the bioherbicide (20 kg fed^−1^) from the three studied cultivars were rapidly frozen in liquid nitrogen, ground, and then homogenized in 100 ml water-soluble extraction buffer. The homogenates were centrifuged for 5 min. at 10,000 rpm, and the clear supernatants were transferred to new tubes. Protein was determined in all samples according to the method suggested by Bradford^34^. The vertical slab polyacrylamide gel electrophoresis (PAGE) was carried out according to the method described by Laemmli^35^ using Mini-gel electrophoresis (BioRad, USA) to determine the relative molecular weights of the isolated proteins. The gel was run in a buffer containing Tris (24 mM) and glycine (194 mM) at room temperature. After completing the electrophoretic run, protein bands were visualized by staining with Commassie Brilliant Blue G-250 and destained overnight with 7% (v/v) glacial acetic acid after documentation^36^. The molecular weights of the separated proteins were estimated by comparing them to standard molecular weight markers specially designed for quantitative and size determination. Protein ladder is a ready-to-use protein molecular weight marker, specially designed for easy quantification and size determination. A Bio-RAD broad marker was used, featuring regularly spaced bands ranging from 6.458 to 195.755 KDa, with 5 µl of the marker loaded in the first well along with the samples.

The polyacrylamide gel plates were photographed, scanned and then analyzed using Quantity One software (Version 4.6.2). This software application is designed for analyzing relative motilities, molecular weights, and amounts of peptide chains as well as providing scanned graphical presentation of the fractionated bands from each lane.

## Results

### Genotoxicity of Si-NPs on Egyptian clover

Genomic template stability (GTS) value (%) was applied to evaluate genotoxicity of applied doses of Si-NPs (14, 22, and 30 g fed^−1^) on Egyptian clover (Helali Cultivar). Results show that GTS value was 100% in Helali cultivar in case of untreated control as well as treatment with the three applied doses (Table 3). This indicates high genome stability of Helali cultivar under applied Si-NPs doses.

**Table 3:** Genomic template stability (GTS) % for each random primer for Si-NPs treatments in Egyptian clover (Helaly cultivar).

	Si-NPs doses			
	Untreated Control	14 g fed^−1^	22 g fed^−1^	30 g fed^−1^
Polymorphic bands found in each treated group	0.00	0.00	0.00	0.00
Average number of polymorphic bands found in each treated group (a)	0.00	0.00	0.00	0.00
Number of total bands in the control sample (n)	25	25	25	25
a/n	0.00	0.00	0.00	0.00
1 − a/n	1	1	1	1
GTS (%) (1 − a/n) * 100	100	100	100	100

### Effect of Egyptian clover cultivars, dodder control treatments, and their interaction on agronomic traits of dodder and Egyptian clover

#### Dodder biomass

Results in Table 4 show that different clover cultivars had a significant effect (*p* ≤ 0.05) on the reduction of fresh and dry weights of dodder under infestation conditions. The Helali cultivar exhibited the most substantial reduction in dodder biomass in all cuttings in both seasons compared to the Gemmiza 1 and Giza 6 cultivars.

**Table 4:** Effect of Egyptian clover cultivars and dodder control treatments on fresh and dry weights of dodder in the 2021/2022 and 2022/2023 seasons.

Cultivars/treatments	Rate fed^−1^	Fresh and dry weights of dodder (g m^−2^)							
		First cut		Second cut		Third cut		Fourth cut	
		FW	DW	FW	DW	FW	DW	FW	DW
2021/2022 Season									
Gemmiza 1		35.3^b^	9.0^a^	62.3^b^	18.5^b^	234.8^b^	53.2^b^	324.8^b^	61.1^b^
Helali		28.9^c^	7.6^b^	51.3^c^	14.9^c^	218.2^c^	49.6^c^	308.2^c^	57.5^c^
Giza 6		40.6^a^	9.6^a^	65.3^a^	19.5^a^	239.2^a^	54.2^a^	329.3^a^	62.1^a^
LSD_0.05_		**1.94**	**0.65**	**1.66**	**0.74**	**2.49**	**0.81**	**2.48**	**0.81**
Bioherbicide	10 kg	51.9^b^	11.1^b^	68.0^b^	19.0^b^	372.0^b^	72.5^b^	542.0^b^	93.9^b^
Bioherbicide	20 kg	44.8^c^	9.7^c^	59.6^c^	16.1^c^	359.4^c^	69.6^c^	529.4^bc^	91.0^c^
Bioherbicide	30 kg	36.9^d^	7.8^d^	50.4^d^	14.4^c^	347.2^d^	67.6^c^	517.2^c^	89.1^c^
Si-NPs	14 g	21.3^e^	3.6^e^	35.4^e^	10.1^d^	114.8^e^	36.5^d^	159.8^d^	34.2^d^
Si-NPs	22 g	16.7^f^	2.4^f^.	32.4^e^	8.7^d^	109.7^e^	35.2^d^	154.7^d^	32.9^d^
Si-NPs	30 g	11.8^g^	1.7^f^	30.6^e^	8.3^d^	106.8^e^	34.7^d^	151.8^d^	32.4^d^
Glyphosate	33.1 g	16.7^f^	1.9^f^	32.3^e^	9.1^d^	110.1^e^	35.5^d^	155.6^d^	33.2^d^
Infested		114.6^a^	40.9^a^	228.8^a^	72.8^a^	556.5^a^	119.2^a^	676.5^a^	135.3^a^
Non-infested		0.0^h^	0.0^g^	0.0^f^	0.0^e^	0.0^f^	0.0^e^	0.0^e^	0.0^e^
LSD_0.05_		**1.84**	**0.80**	**7.05**	**1.99**	**11.81**	**2.06**	**15.66**	**2.00**
2022/2023 Season									
Gemmiza 1		43.2^b^	11.3^b^	106.3^b^	21.1^b^	258.0^b^	46.8^b^	371.2^b^	69.9^b^
Helali		35.3^c^	9.6^c^	87.6^c^	17.0^c^	250.1^c^	42.6^c^	351.8^c^	65.7^c^
Giza 6		49.6^a^	12.1^a^	112.2^a^	22.3^a^	274.2^a^	47.9^a^	376.2^a^	71.0^a^
LSD_0.05_		**2.37**	**0.74**	**2.91**	**0.89**	**5.04**	**0.94**	**3.70**	**0.94**
Bioherbicide	10 kg	63.3^b^	14.5^b^	116.7^b^	21.3^b^	425.7^b^	67.1^b^	620.3^b^	107.4^b^
Bioherbicide	20 kg	54.6^c^	12.6^c^	102.2^c^	18.4^bc^	411.3^b^	63.7^c^	605.9^bc^	104.0^bc^
Bioherbicide	30 kg	45.0^d^	9.3^d^	86.4^d^	16.4^c^	397.2^b^	61.5^c^	591.8^c^	101.8^c^
Si-NPs	14 g	26.1^e^	4.6^e^	60.9^e^	11.5^d^	131.2^c^	26.0^d^	182.9^d^	39.2^d^
Si-NPs	22 g	20.4^f^	3.2^f^	55.0^e^	10.0^d^	125.7^c^	24.4^d^	177.0^d^	37.6^d^
Si-NPs	30 g	14.4^ g^	2.2^f^	51.6^e^	9.5^d^	122.3^c^	23.9^d^	173.6^d^	37.1^d^
Glyphosate	33.1 g	20.4^f^	2.5^f^	55.3^e^	10.4^d^	126.0^c^	24.8^d^	177.3^d^	38.0^d^
Infested		140.2^a^	50.0^a^	390.2^a^	83.3^a^	606.8^a^	120.6^a^	768.8^a^	154.8^a^
Non-infested		0.0^h^	0.0^g^	0.0^f^	0.0^e^	0.0^d^	0.0^e^	0.0^e^	0.0^e^
LSD_0.05_		**2.74**	**1.08**	**14.22**	**3.12**	**31.01**	**3.39**	**25.12**	**3.74**

FW = Fresh weight, DW = dry weight. Values (means) followed by different letter(s) are significantly different according to LSD at *p* ≤ 0.05. LSD values are in bold.

Similarly, significant differences (*p* ≤ 0.05) were found among different control treatments under dodder infestation conditions (Table 4). All weed control treatments significantly decreased the dodder biomass as compared to the infested control in both seasons. In general, Si-NPs treatments and glyphosate were the most effective (often showing insignificant differences between them), followed by bioherbicide treatments. Specifically, Si-NPs at 30 g fed^−1^ exerted the highest significant reduction in dodder biomass in the four cuttings during the two seasons in comparison with the infested control. Glyphosate at 33.1 g fed^−1^ came in the second rank.

Among the bioherbicide treatments, the 30 kg fed^−1^ was the most effective in reducing dodder biomass in all cuttings, although it was not significantly different from the 20 kg fed^−1^ rate in some cuttings during both seasons (Table 4).

Data presented in Supplementary Table 1 illustrate the effect of interactions between Egyptian clover cultivars and dodder control treatments on the fresh and dry weights of dodder. Significant differences (*p* ≤ 0.05) were noted among cultivars and treatments for dodder biomass across four consecutive cuttings in both seasons. The most effective interaction for reducing dodder biomass was achieved with Helali cultivar when treated with Si-NPs at either 22 or 30 g fed^−1^, showing no significant differences among them. Nevertheless, the highest reduction in dodder biomass was obtained with the combination of Helali cultivar and Si-NPs treatment at 30 g fed^−1^ in the four cuttings.

#### Egyptian clover biomass

Data shown in Table 5 reveal that the three Egyptian clover cultivars significantly influenced (*p* ≤ 0.05) the fresh and dry weights of clover under dodder infestation conditions. The Helali cultivar yielded the highest significant mean values for clover biomass in all cuttings during both seasons compared to Gemmiza 1 and Giza 6 cultivars.

**Table 5:** Effect of Egyptian clover cultivars and dodder control treatments on fresh and dry weights of clover in the 2021/22 and 2022/23 seasons.

Varieties/treatments	Rate fed^−1^	Fresh and dry weights of clover (ton fed^−1^)							
		First cut		Second cut		Third cut		Fourth cut	
		FW	DW	FW	DW	FW	DW	FW	DW
2021/2022 Season									
Gemmiza 1		7.20^b^	1.24^ab^	11.42^b^	1.78^b^	11.02^b^	1.77^b^	10.95^b^	1.74^b^
Helali		7.54^a^	1.31^a^	11.53^a^	1.88^a^	11.56^a^	1.87^a^	11.63^a^	1.84^a^
Giza 6		6.82^c^	1.20^b^	10.84^c^	1.77^b^	10.85^b^	1.74^b^	10.72^b^	1.72^c^
LSD_0.05_		**0.044**	**0.007**	**0.043**	**0.023**	**0.171**	**0.034**	**0.533**	**0.013**
Bioherbicide	10 kg	6.41^d^	1.15^d^	9.96^e^	1.67^e^	9.05^e^	1.53^d^	8.47^e^	1.40^d^
Bioherbicide	20 kg	6.78^c^	1.19^c^	10.63^d^	1.75^d^	9.68^d^	1.6^c^	9.06^d^	1.47^c^
Bioherbicide	30 kg	6.84^c^	1.19^c^	10.77^d^	1.77^c^	9.83^d^	1.62^c^	9.15^d^	1.48^c^
Si-NPs	14 g	7.60^b^	1.32^b^	11.90^c^	1.91^b^	12.44^c^	1.97^b^	12.72^c^	2.02^b^
Si-NPs	22 g	7.64^b^	1.33^b^	12.17^b^	1.95^a^	12.77^b^	2.01^a^	13.01^b^	2.05^a^
Si-NPs	30 g	7.69^b^	1.34^b^	12.21^b^	1.95^a^	12.78^b^	2.01^a^	13.08^b^	2.05^a^
Glyphosate	33.1 g	7.66^b^	1.33^b^	12.17^b^	1.94^a^	12.74^b^	2.01^a^	12.99^b^	2.05^a^
Infested		5.64^e^	1.01^e^	7.76^f^	1.44^f^	7.68^f^	1.37^e^	7.57^f^	1.30^e^
Non-infested		8.44^a^	1.37^a^	12.66^a^	1.95^a^	13.31^a^	2.02^a^	13.83^a^	2.08^a^
LSD_0.05_		**0.233**	**0.020**	**0.162**	**0.019**	**0.167**	**0.026**	**0.187**	**0.036**
2022/2023 Season									
Gemmiza 1		6.10^b^	1.09^b^	9.69^b^	1.56^b^	9.67^b^	1.55^b^	9.61^b^	1.53^b^
Helali		6.39^a^	1.15^a^	10.12^a^	1.65^a^	10.15^a^	1.64^a^	10.21^a^	1.61^a^
Giza 6		5.78^c^	1.05^c^	9.51^c^	1.56^b^	9.53^b^	1.53^b^	9.41^b^	1.51^c^
LSD _0.05_		**0.037**	**0.007**	**0.038**	**0.022**	**0.152**	**0.030**	**0.469**	**0.013**
Bioherbicide	10 kg	5.45^d^	1.01^e^	8.77^e^	1.47^e^	7.97^e^	1.35^d^	7.46^e^	1.23^d^
Bioherbicide	20 kg	5.76^c^	1.04^d^	9.36^d^	1.54^d^	8.52^d^	1.41^c^	7.98^d^	1.29^c^
Bioherbicide	30 kg	5.81^c^	1.05^d^	9.48^d^	1.56^c^	8.65^d^	1.42^c^	8.05^d^	1.31^c^
Si-NPs	14 g	6.46^b^	1.16^c^	10.47^c^	1.68^b^	10.95^c^	1.74^b^	11.19^c^	1.78^b^
Si-NPs	22 g	6.50^b^	1.17^bc^	10.71^b^	1.71^a^	11.24^b^	1.77^a^	11.45^b^	1.81^a^
Si-NPs	30 g	6.54^b^	1.18^b^	10.75^b^	1.71^a^	11.24^b^	1.77^a^	11.51^b^	1.81^a^
Glyphosate	33.1 g	6.51^b^	1.17^bc^	10.71^b^	1.71^a^	11.21^b^	1.77^a^	11.44^b^	1.80^a^
Infested		4.63^e^	0.85^f^	6.59^f^	1.22^f^	6.53^d^	1.17^e^	6.44^f^	1.10^e^
Non-infested		7.18^a^	1.21^a^	11.15^a^	1.71^a^	11.71^a^	1.78^a^	12.17^a^	1.83^a^
LSD_0.05_		**0.198**	**0.018**	**0.142**	**0.017**	**0.146**	**0.022**	**0.164**	**0.033**

FW = Fresh weight, DW = dry weight. Values (means) followed by different letter(s) are significantly different according to LSD at *p* ≤ 0.05. LSD values are in bold.

A significant decrease (*p* ≤ 0.05) was noted in the fresh and dry weights of dodder-infested clover plants compared to non-infested ones in the four cuttings during both seasons (Table 5).

Weed control treatments showed also a significant effect (*p* ≤ 0.05) on the fresh and dry weights of clover under infestation conditions (Table 5). All tested concentrations of Si-NPs and glyphosate at 33.1 g fed^−1^ significantly increased clover biomass compared to the infested control, with most cuttings showing no significant differences among these treatments during both growing seasons. Si-NPs at 30 g fed^−1^ maintained the highest increment in fresh weight in the four cuttings during the two seasons, while the highest increment in dry weight was equally sustained by Si-NPs at 22 and 30 g fed^−1^ for the second, third, and fourth cuttings in both seasons, respectively, compared to the infested control.

In a similar manner, bioherbicide treatment at 20 and 30 kg fed^−1^ significantly increased clover biomass for most cuttings in the first and second seasons under infestation conditions (Table 5). However, the bioherbicide treatment at 30 kg fed^−1^ outperformed the 20 kg fed^−1^ rate.

Results of the interaction effect between Egyptian clover cultivars and dodder control treatments on clover’s fresh and dry weights showed significant differences (*p* ≤ 0.05) in the four consecutive cuttings during both seasons (Supplementary Table 2). The combination of the Helali cultivar with all doses of Si-NPs exhibited the highest significant values for clover biomass in all cuttings during both seasons. The maximum fresh weight was mostly attained with Helali cultivar and Si-NPs treatment at 30 g fed^−1^ during both seasons, while the maximum dry weight was obtained with the combination of Helali cultivar with Si-NPs at 22 g fed^−1^ for the second, third and fourth cuttings in both seasons. Worthy to note that the mean values obtained from this interaction were not significantly different from those recorded for the non-infested control plants of the Helali cultivar for the first cutting of the first season and the second, third and fourth cuttings during both consecutive seasons.

#### Egyptian clover yield parameters

A significant effect (*p* ≤ 0.05) of Egyptian clover cultivars was exhibited on seed yield parameters under dodder infestation conditions (Table 6). Results show that Helali cultivar was the most effective in increasing the number of seeds/head, 1000 seed weight, and overall seed yield compared to Gemmiza 1 and Giza 6 cultivars.

**Table 6:** Effect of Egyptian clover cultivars and dodder control treatments on seed yield parameters of clover in the 2021/2022 and 2022/2023 seasons.

Cultivars/treatments	Rate fed^−1^	No. seeds head^−1^		1000 Seed weight (g)		Seed yield (kg fed^−1^)	
		1st Season	2nd Season	1st Season	2nd Season	1st Season	2nd Season
Gemmiza 1		45.07^b^	47.37^b^	3.05^a^	3.21^a^	309.8^b^	325.5^b^
Helali		48.74^a^	51.26^a^	2.92^b^	3.07^b^	323.6^a^	340.1^a^
Giza 6		41.96^c^	44.07^c^	2.86^b^	3.00^b^	286.4^c^	300.4^c^
LSD_0.05_		**1.62**	**1.74**	**0.071**	**0.100**	**10.84**	**11.61**
Bioherbicide	10 kg	38.9^d^	41.0^e^	2.48^f^	2.60^d^	263.1^d^	276.2^de^
Bioherbicide	20 kg	46.2^c^	48.6^d^	3.09^d^	3.26^bc^	312.9^c^	328.4^c^
Bioherbicide	30 kg	39.8^d^	41.9^e^	2.62^e^	2.76^d^	267.8^d^	281.1^d^
Si-NPs	14 g	46.6^c^	49.0^cd^	3.02^d^	3.19^c^	315.7^c^	331.4^c^
Si-NPs	22 g	49.8^b^	52.2^b^	3.25^b^	3.42^a^	334.5^b^	350.8^b^
Si-NPs	30 g	48.3^b^	50.8^bc^	3.15^c^	3.30^b^	329.5^b^	345.8^b^
Glyphosate	33.1 g	48.4^b^	50.8^bc^	3.19^c^	3.34^b^	332.4^b^	349.1^b^
Infested		36.6^e^	38.6^f^	2.34^g^	2.47^e^	253.6^e^	268.1^e^
Non-infested		52.8^a^	55.3^a^	3.35^a^	3.52^a^	349.9^a^	366.9^a^
LSD_0.05_		**1.68**	**2.12**	**0.052**	**0.109**	**7.09**	**11.23**

1st Season = 2021/2022 season, 2nd Season = 2022/2023 season. Values (means) followed by different letter(s) are significantly different according to LSD at *p* ≤ 0.05. LSD values are in bold.

Seed yield parameters were significantly lower in infested plants compared to non-infested ones (Table 6). Decrements were recorded for number of seeds/head (30.7 and 30.2%), 1000 seeds weight (30.1 and 29.8%), and seed yield (27.5 and 26.9%) for infested plants in the two respective seasons relative to non-infested plants.

Significant differences in seed yield parameters were also evident with the application of different control treatments under dodder infestation conditions (Table 6). Although Si-NPs at 22 and 30 g fed^-1^ and glyphosate at 33.1 g fed^−1^ were equally effective in increasing seed yield parameters, Si-NPs at 22 g fed^−1^ had the highest significant effect compared to the infested control.

Among the bioherbicide treatments, the application of bioherbicide at 20 kg fed^−1^ had the most significant effect on seed yield parameters under infestation conditions (Table 6).

Data presented in Supplementary Table 3 show that the interaction between Egyptian clover cultivars and dodder control treatments was significant (*p* ≤ 0.05) for the number of seeds/head, seed yield and weight of 1000 seeds in both seasons. The highest number of seeds/head was observed with the interaction of Helali cultivar and Si-NPs at 22 g fed^−1^ in both seasons. The most effective interaction for enhancing the weight of 1000 seeds was noted between Gemmiza 1 cultivar and Si-NPs treatment at 22 g fed^−1^ in both seasons. Other significant interactions that improved 1000 seed weight included Gemmiza 1 with glyphosate, Gemmiza 1 with Si-NPs at 30 g fed^−1^ and Helali with Si-NPs at 22 g fed^−1^.

In terms of seed yield, the highest significant values were recorded for the interactions between Helali and glyphosate, Gemmiza 1 and Si-NPs at 22 g fed^−1^, Helali and Si-NPs at 22 g fed^−1^, and Helali and Si-NPs at 30 g fed^−1^. Notably, these interactions showed no significant differences among them.

### Effect of dodder infestation and bioherbicide treatment on clover stem anatomy

**Fig. 3:**
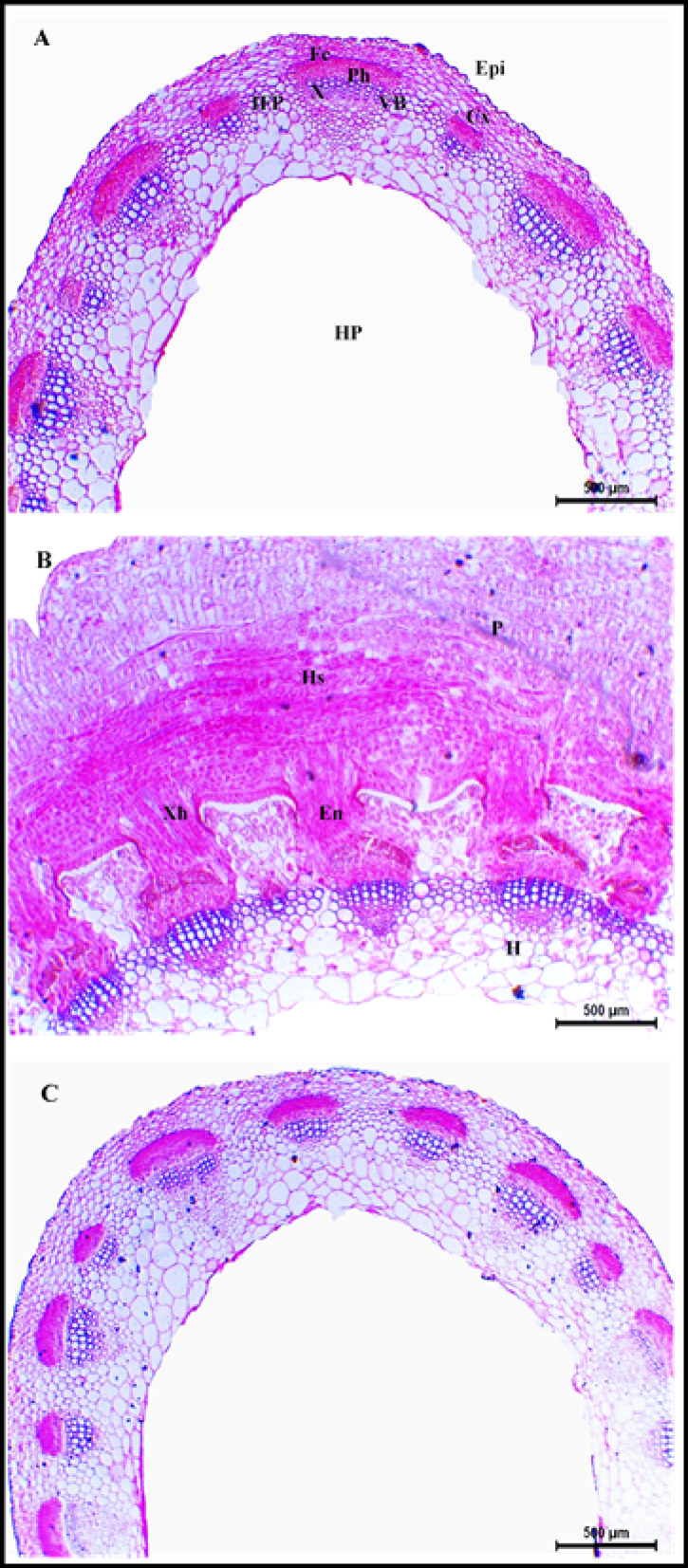
Transverse sections of Egyptian clover stem (Helali cultivar). (**A**) Control, (**B**) dodder infested, (**C**) dodder infested-bioherbicide treated (20 kg fed^-1^). Epi: epidermis, Cx: cortex, IFP: interfascicular parenchyma, VB: vascular bundle, Fc: fiber cap, Ph: phloem X: xylem, HP: hollow pith, P: Parasite, Hs: haustorium, En: endophyte, Xh: xylem hyphae, H: host tissues. Scale bar = 500 µm.

**Fig. 4:**
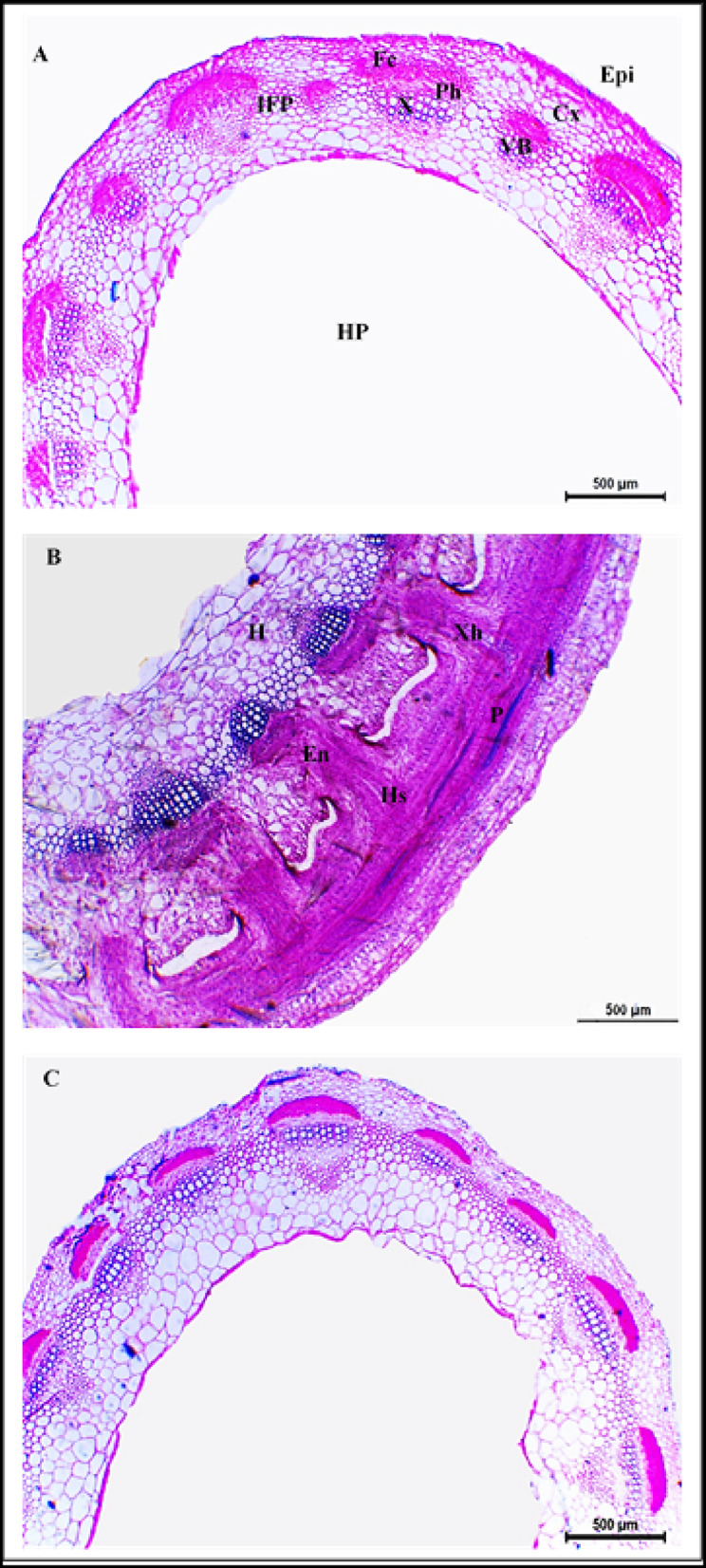
Transverse sections of Egyptian clover stem (Giza 6 cultivar). (**A**) Control, (**B**) dodder infested, (**C**) dodder infested-bioherbicide treated (20 kg fed^-1^). Epi: epidermis, Cx: cortex, IFP: interfascicular parenchyma, VB: vascular bundle, Fc: fiber cap, Ph: phloem, X: xylem, HP: hollow pith, P: Parasite, Hs: haustorium, En: endophyte, Xh: xylem hyphae, H: host tissues. Scale bar = 500 µm.

**Fig. 5:**
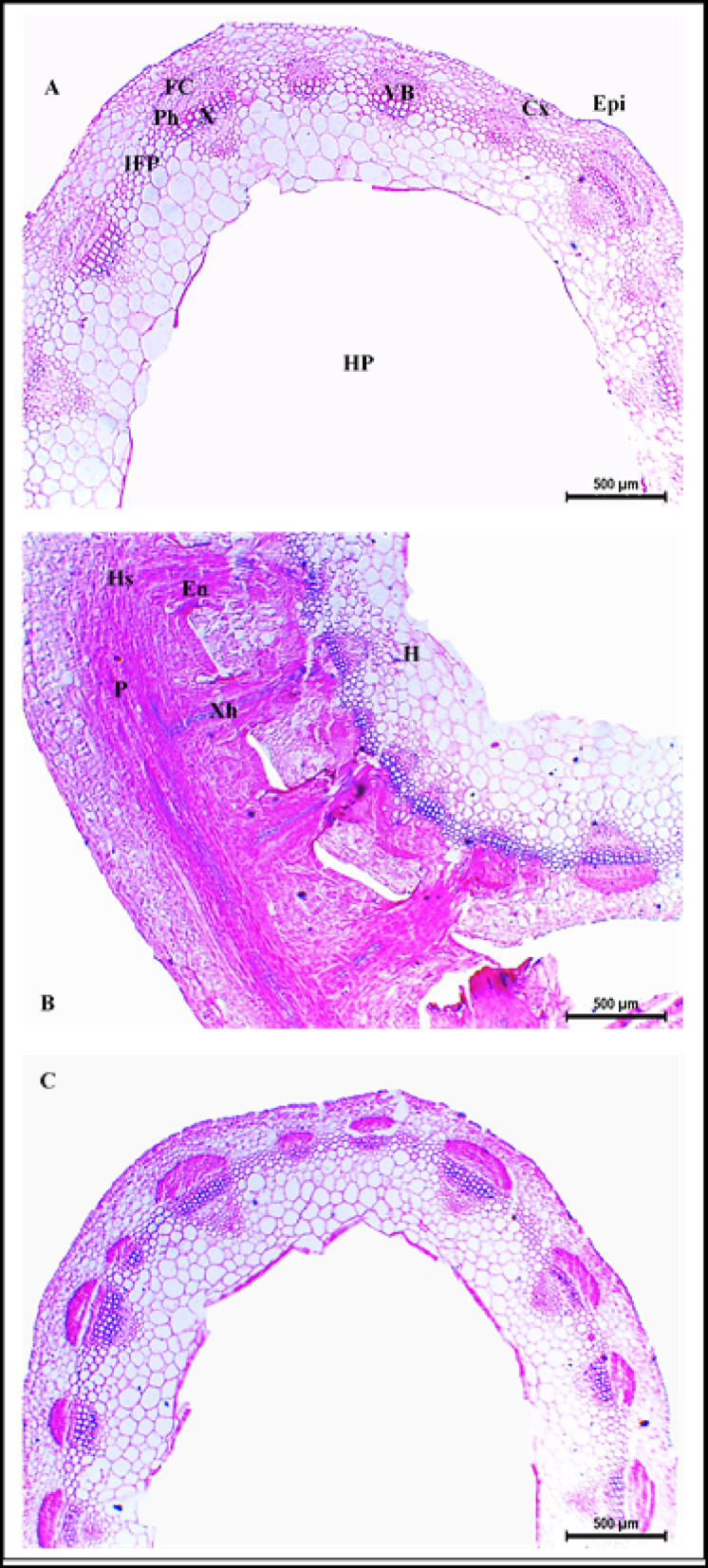
Transverse sections of Egyptian clover stem (Gemmiza 1 cultivar). (**A**) Control, (**B**) dodder infested, (**C**) dodder infested- bioherbicide treated (20 kg fed^-1^). Epi: epidermis, Cx: cortex, IFP: interfascicular parenchyma, VB: vascular bundle, Fc: fiber cap, Ph: phloem, X: xylem, HP: hollow pith, P: Parasite, Hs: haustorium, En: endophyte, Xh: xylem hyphae, H: host tissues. Scale bar = 500 µm.

Stems infested with dodder (*C. planiflora*) exhibited similar structural changes for all three cultivars, where the haustoria of *C. planiflora* clearly penetrated the clover stem’s epidermis and cortex, establishing connections with the vascular bundles (Figs. 3, 4 and 5). Formation of xylem-connecting hyphae and the disrupted shape of vascular bundles were more distinct in the stems of Gemmiza 1 cultivar compared to those of the Helali and Giza 6 (Figs. 3, 4 and 5). Moreover, the haustorial penetration caused an apparent disruption of the epidermal and cortical layers, which was associated with hypertrophy of cortical cells (Figs. 3, 4 and 5). Such increase in the size of cortical cells resulted in thicker cortical layer and stem wall with relative increases of 101.2 and 91.5% for Helali, 65 and 109.9% for Giza 6, and 5.3 and 68.7% for Gemmiza 1 relative to their controls (Supplementary Table 4). Additionally, xylem vessels of the infested stems were compactly arranged and showed increased lignification (Figs. 3, 4 and 5).

In contrast, the anatomical structure of plants of the three studied cultivars infested with dodder and treated with bioherbicide (20 kg fed^−1^) showed no signs of dodder stems or haustoria either attached or close to clover stems. Moreover, the stem structural arrangement and histological measurements of these treated plants were similar to those of the healthy control plants of the three clover cultivars (Supplementary Table 4, Figs. 3, 4 and 5).

### Electrophoretic protein pattern

**Fig. 6:**
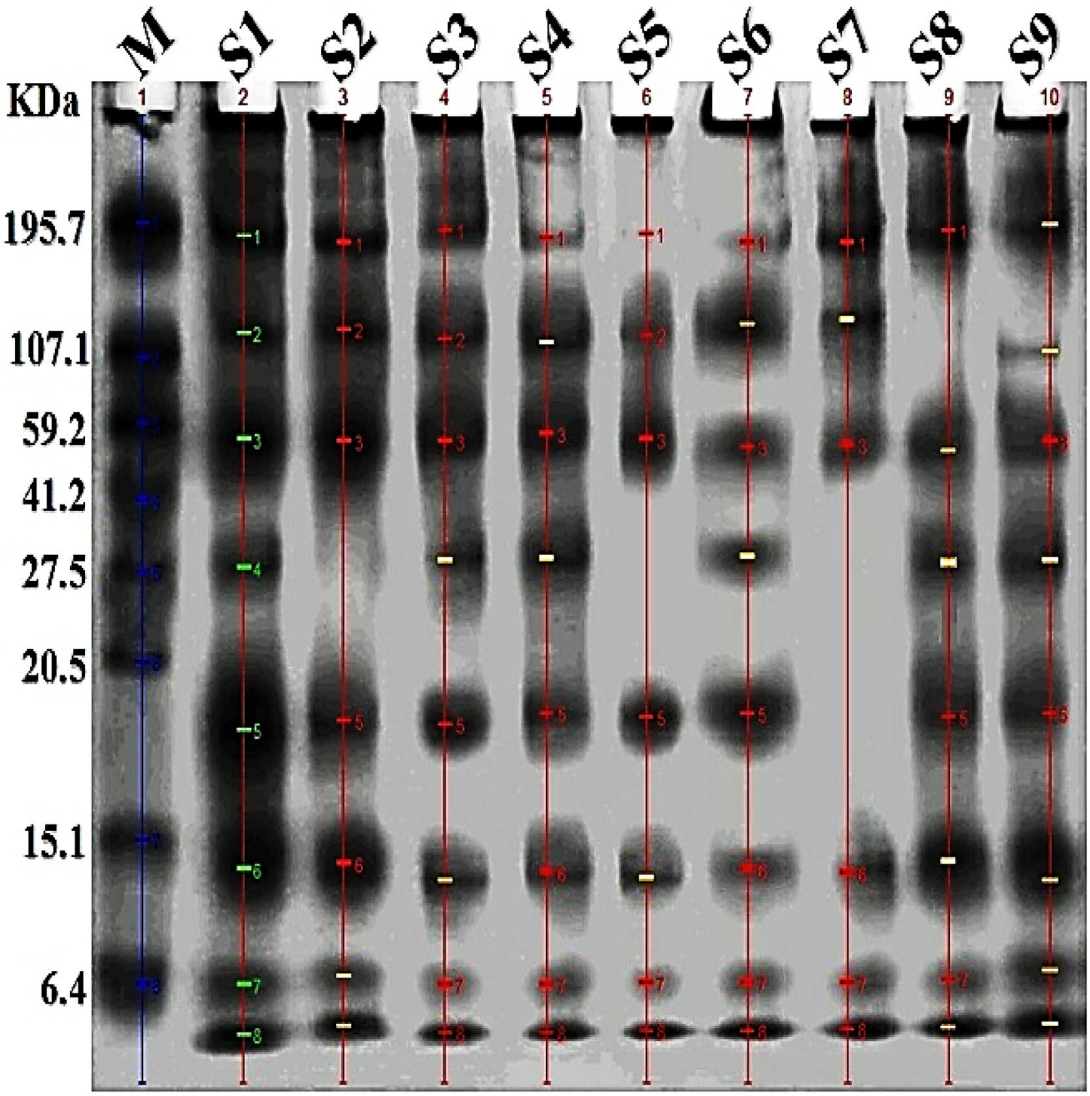
Electrophoretic protein pattern by SDS-PAGE, M is molecular weight marker with 8 bands of known sizes ranging from 6.4 -195.7 KDa. **S1:** Gemmiza 1 (healthy control), **S2:** Giza 6 (healthy control), **S3:** Helali (healthy control), **S4:** Helali; dodder infested-bioherbicide treated (20 kg fed^-1^), **S5:** Gemmiza 1; dodder infested-bioherbicide treated (20 kg fed^-1^), **S6:** Giza 6; dodder infested-bioherbicide treated (20 kg fed^-1^). **S7:** Gemmiza 1; dodder infested, **S8:** Giza 6; dodder infested, **S9:** Helali; dodder infested.

Supplementary Table 5 and Fig. 6 present the SDS-Page protein analysis of three cultivars of Egyptian clover (Helali, Giza 6, and Gemmiza 1) under three conditions: as controls (healthy plants), plants infested with dodder, and plants infested with dodder and treated with *F. incarnatum*-based bioherbicide (20 kg fed^−1^).

The protein analysis of the three studied cultivars showed many shared bands among the three cultivars, with a few unique bands associated with specific cultivars and/or treatments. The positive sign (+) refers to presence of a band, whereas, negative sign (−) denotes its absence (Supplementary Table 5). The common bands across all conditions (healthy controls, infested plants, and infested plants treated with the bioherbicide) were identified at molecular weights of 184.41, 55.06, 12.75, 6.40, and 4.72 KDa. Notably, bands were absent at 119.60 KDa for infested Giza 6, 28.09 KDa for the control of Giza 6, as well as for infested and infested-treated Gemmiza 1, and at 18.24 KDa for infested Gemmiza 1 (Supplementary Table 5, Fig. 6).

### Economic evaluation of Si-NPs and *Fusarium* spp. as potential herbicides

The data presented in Table 7 suggest that Si-NPs and *Fusarium* bioherbicide are highly competitive with conventional chemical herbicides in terms of treatment costs and number of applications required per season. Moreover, total fresh yield of clover is comparable for both Si-NPs and conventional bioherbicide treatments (based on the results of the current study), surpassing that produced with bioherbicide treatment. The highest net income was achieved with the application of Si-NPs, exceeding that obtained with conventional herbicide, while *Fusarium* bioherbicide came in third rank.

**Table 7:** Hypothetical cost–benefit comparison of Si-NPs and *Fusarium*-based granular bioherbicide versus conventional herbicide use in Egyptian clover production (per feddan*).

Parameter	Si-NPs as herbicides	*Fusarium* bioherbicide	Conventional herbicide
Material cost (EGP)	300–700 (local source)	250 (local source)	750–900 (imported)
Cost/application (EGP)	150	150	150
No. of applications	1–2/season	1/season	2–3/season
Total application cost (EGP)	300	150	450
Total treatment cost (EGP)	600–1000	400	1200–1350
Clover fresh yield (ton/feddan*/season)	42.9	34.3	42.7
Clover market price (EGP/ton FW)	2000	2000	2000
Gross income (EGP)	85,800	68,600	85,400
Net income (EGP) after deducting treatment cost	85,200–84,800	68,200	84,200–84,050

*1 feddan =  ~ 0.42 hectares, 1 EGP = 0.020 USD (May 2025).

Average fresh yield per season was calculated for each treatment based on the results achieved in the current study.

Market prices are based on 2024 average values from Delta region reports. Actual figures may vary depending on market fluctuations and yield conditions.

## Discussion

### Effect of dodder parasitism on clover productivity

Egyptian clover (*T. alexandrinum* L.) is a very important forage crop in Egypt owing to its vital role in sustaining high quality fodder for livestock, either as green feed or in conserved forms such as hay or silage^2^. As a leguminous plant, it contributes significantly to soil fertility and helps preventing its erosion during crop rotations^2^. This is essential for sustaining high agricultural crop productivity under the conditions of diminishing arable land and increased competition between strategic crops such as wheat, maize, and rice^2^. Dodder (*C. planiflora*) is the major parasitic weed that adversely affects the productivity of Egyptian clover^1^. The yield losses caused by *Cuscuta* spp. can be as high as 80%^5^. The current study reported significant decrements in forage and seed yields of dodder-infested Egyptian clover. This finding aligns with previous research reporting reductions in forage and seed yields of Egyptian clover because of dodder infestation^1,3,7–9^. Additionally, Saric-Krsmanovic et al.^37^ noted reductions in fresh weight of alfalfa, a closely related legume and forage crop, due to dodder infestation.

### Management of dodder infestation in Egyptian clover

#### Resistant/tolerant cultivars

According to Qasem^5^, managing parasitic weeds such as dodder involves a multifaceted approach, including the selection of resistant and/or tolerant genotypes/cultivars. In the case of Egyptian clover, a previous study showed that cultivars such as Helali, Gemmiza 1, Giza 6, and Sakha 4 have shown greater tolerance to dodder, evidenced by lower infestation rates and decreased forage yield losses in multiple cuts over two growing seasons^1^. These results were the foundation for the choice of the three cultivars: Helali, Gemmiza 1, and Giza in the current investigation. Another report^3^ specifically highlighted the Helali cultivar for its resilience against dodder, showing less reduction in forage yield under infestation conditions^3^. The current study’s findings support the notion that the Helali cultivar is more competitive with other cultivars against dodder infestation. That was evidenced by the highest significant reductions in dodder biomass, alongside the least significant reductions in forage and seed yields under infestation conditions.

This high level of tolerance of the Helali cultivar to dodder infestation could be attributed to several factors. The key factor that was highlighted by El-Refaey et al.^11^ is its higher content of phenolic compounds (~ 1111.65 µg 100 mg^−1^ foliage dry weight) compared to other clover genotypes. Phenolic compounds are secondary plant metabolites that play a critical role in the plant’s defense mechanisms against environmental stresses^38^. Lignin is another important secondary metabolite that enhances resistance to parasitic invasion of dodder via the reinforcement of host plant cell walls, as evidenced by Zhou et al.^38^ in white clover. Parasitic invasion of dodder through haustorial penetration stimulates defense pathways and induces the production of defense phytohormones such as salicylic acid (SA), jasmonic acid (JA), and abscisic acid (ABA)^38^. Moreover, genetic diversity in the Helali cultivar compared to others such as Gemmiza 1, Giza 6, Sakha 4, and Serw 1 has also been documented^10^. However, further investigation is required to explore the role of this genetic variation in inducing tolerance to dodder invasion.

#### Chemical herbicides

Chemical herbicides, particularly glyphosate, have been extensively used over decades for effective control of dodder^5,8,12,13,37^. In the present study, glyphosate application resulted in reduced dodder growth parameters compared to dodder-infested control. Glyphosate interferes with the shikimic acid pathway in target weeds, affecting the biosynthesis of defensive compounds such as phytoalexins and flavonoids^13,37^. However, the excessive use of such herbicides poses risks to the environment and human health with increased potential of carcinogenicity, added to higher costs of application per unit area^14^. The drawbacks associated with chemical herbicides have urged the need for alternative control methods, which are safer to the environment and more cost effective.

#### Si-NPs as alternatives to chemical herbicides

Si-NPs have emerged in the recent time as promising alternatives to traditional herbicides^15,16^. Si-NPs possess several advantageous properties, including a high surface area, porosity, penetrability, and biochemical reactivity, all while exhibiting low environmental toxicity^16,17,39^. These characteristics enable Si-NPs to be used either directly as herbicides or as nano-carriers for enhancing the efficacy of other herbicides^16^.

While the mechanisms of Si and Si-NPs in controlling pathogenic attacks have been extensively investigated, research on their practical effects in weed control, especially *Cuscuta*, remains limited. However, Al-Gburi^21^ reported that Si mitigated the adverse effects of *C. campestris* on eggplant, improving its physiological parameters through enhanced activity of antioxidant enzymes, phenolic compounds, and defense hormones such as salicylic acid. Similarly, Lukacova et al.^20^ found that Si effectively counteracted dodder infestation in tobacco, leading to improved growth parameters and reduced content of antioxidant enzymes such as CAT, POD, and SOD. The current research aligns with the existing literature, reporting decrements in dodder fresh and dry biomass, in response to treatment with Si-NPs in comparison with infested control. The best results were achieved at higher application rates of 22 and 30 g fed^−1^, which were comparable to, if not exceeding the effect of glyphosate applied at 33.1 g fed^−1^. Moreover, the productivity of Egyptian clover was significantly improved in all cuttings during both seasons with these same Si-NPs doses as compared to infested control.

Exploring the action modes of Si-NPs against pests and weeds, it was found that they act in the host plant through two primary mechanisms: physico-mechanical and biochemical/molecular^16–19^. Upon penetrating plant tissues, silicon accumulates in cell walls, forming non-crystalline structures known as phytoliths, which enhance cell rigidity and act as physical barriers against invading pathogens^16–19,39,40^. In addition to their physical roles, Si-NPs also activate several biochemical mechanisms that enable them to counteract biotic pathogens. For example, Si has a vital role in enhancing the gene expression and activity of defensive antioxidant enzymes such as chitinase, peroxidase (POD), catalase (CAT), superoxide dismutase (SOD), phenylalanine ammonia lyase (PAL), and polyphenol oxidase (PPO)^16,18,19,39,40^. These antioxidant enzymes are crucial for mitigating oxidative stress caused by pathogens and the overproduction of reactive oxygen species (ROS)^16^. PAL, for example, is particularly important in the production of phenolic compounds, serving as a key enzyme in the phenylpropanoid metabolic pathway and a precursor for the synthesis of defensive compounds such as lignin and phytoalexins^18,39,40^. Phytoalexins are antimicrobial compounds produced by plants in response to pathogenic attacks, while lignin reinforces cell walls, acting as a physical barrier against pathogen invasion^40^. Similarly, PPO and POD play an important role in polymerization of phenols and consequently accumulation of lignin^18,40^. PPO is also capable of oxidizing phenols into quinines, which are more toxic to pathogens^40^. Si-NPs are also vital for the induction of systemic acquired resistance (SAR) through mediation of systemic signals such as salicylic acid (SA), jasmonic acid (JA), abscisic acid (ABA), and ethylene, which are transmitted throughout plant tissues to enhance defense against stress conditions^16–19^.

Improvements in clover productivity under dodder infestation can be attributed to the dual role of Si-NPs in controlling dodder and enhancing plant growth. Silicon facilitates the absorption and translocation of essential macro-elements such as Ca, Mg, and K, which are vital for improving plant integrity under stress conditions. This enhances the general mineral status, photosynthetic activity, and overall plant development^17,19,41,42^. Si also helps plants to retain water content and suppress the production of ethylene, thereby delaying aging and leaf senescence^39^.

This study highlights also the effective interaction between the resistant/tolerant Helali cultivar and the application of Si-NPs as a viable control method for dodder. Utilizing Si-NPs not only provides a sustainable alternative to chemical herbicides but also enhances the overall resilience and productivity of Egyptian clover under parasitic stress.

#### Biological herbicides as alternatives to chemical control

The increasing importance of biological herbicides (bio-control) as alternatives of chemical herbicides for weed control also has been recognized over the last few decades^22^. Fungal species, in particular, are amongst the most widely used natural antagonists in controlling major weed species such as *Striga*, *Orobanche*, and *Cuscuta* ^22–24^. Among these, *Fusarium* species have been frequently reported for their efficacy in managing the aforementioned weeds^22,25,26,43–45^. However, there are limited studies that specifically focus on the effective control of dodder using *Fusarium* spp.^25,44,45^.

The current research reported effective control of dodder, evidenced by significant decrements in dodder biomass in all cuttings during both growing seasons with the application of *F. incarnatum* as a granular bioherbicide at rates of 20 and 30 kg fed^−1^ in comparison with the infested control. Moreover, improvements were achieved in clover productivity during both seasons with the same application rates versus the infested control, reinforcing the efficacy of this control method. Supporting evidence from Fallahpour et al.^25^ indicated the successful control of *C. campestris* in various crops, including sugar beet, alfalfa, basil, wheat and rice, using *F. oxysporum* as a bioherbicide. Furthermore, *F. incarnatum* has also proven effective in the control *C. gronovii* in green and black gram^44,45^.

One of the major advantages of the selected biocontrol agent, *F. incarnatum* is its host specificity, allowing it to target the parasitic weed (dodder) without harming the host plant^27,46,47^. It can exert a direct pathogenic effect on weed species through the colonization of weed vascular tissues or by secreting toxic substances^27,48^. Additionally, being pathogenic in nature, fungal species such as *Fusarium* are capable of inducing systemic resistance in host plants through the activation of systemic signaling pathways, particularly SA and JA, along with enhancing the production of defense-related enzymes and metabolites such as PPO, lignin and phytoalexins^27,47,48^. These mechanisms in addition to the long persistence of *Fusarium* in the soil, further support its effective control of dodder during its early developmental stages (pre-emergence) and subsequent life stages^22,27,48^. Moreover, some *Fusarium* spp. could establish beneficial endophytic relationships with the host plant, improving its resilience to pathogenic attacks^49^.

#### Comparative efficacy of Si-NPs and *F. incarnatum* bioherbicide

While Si-NPs demonstrated a higher efficacy than *F. incarnatum* bioherbicide in controlling dodder, a combined approach utilizing both methods could potentially yield even greater efficacy. Such an integrated strategy could lower costs and minimize environmental risks, making it a sustainable alternative for managing dodder infestations in Egyptian clover and potentially other crops. This research highlights the importance of exploring and integrating biological control methods such as *F. incarnatum* with nanomaterials such as Si-NPs, to enhance the sustainability and effectiveness of weed management strategies in agriculture.

### Effect of dodder infestation and bioherbicide treatment on clover stem anatomy

#### Structure of healthy stem

The anatomy of healthy Egyptian clover stems exhibits a distinct structure. The stem is oval-shaped, with an epidermis comprised of a single layer of cells. Beneath the epidermis, the cortex consists of one layer of collenchymatous cells followed by several layers of parenchyma. Numerous collateral vascular bundles are arranged in a cylindrical pattern, alternating between larger and smaller bundles, separated by interfascicular lignified parenchyma. Each vascular bundle is covered by a sclerenchymatous cap adjacent to the phloem tissue, while the xylem vessels are arranged in radial rows. Some parenchyma layers extend towards the center, leading to a clear hollow pith. This anatomical structure is consistent across the three cultivars of Egyptian clover (Helali, Giza 6, and Gemmiza 1) studied in the current investigation, matching the descriptions provided by Zoric et al.^50^ for some *Trifolium* species.

#### Structure of dodder-infested stem

The anatomical structure of clover stems infested with *C. planiflora* undergoes significant alterations due to dodder haustorial penetration. Haustoria, specialized stem outgrowths of *Cuscuta*, penetrate the host’s stems, acting as transfer channels for nutrients and water between the host and parasite^51,52^. Disruption and dissolution of epidermis at haustorial penetration sites is the first change to be noted^53^. Such deformation is caused by the mechanical pressure induced by haustoria against host cell walls, coupled with the secretion of cell wall-degrading enzymes such as cellulases and pectinases^54^.

Following penetration, the endophyte (lower haustorium) develops into searching hyphae, which seek vascular connections with the host^4,9,55^. Upon connection with vascular elements, searching hyphae will differentiate into either xylem or phloem hyphae, depending on the type of vascular element in connection^9,55^. Moreover, if searching hyphae are not able to penetrate fibrous elements covering the vascular bundle, instead, they will rotate around them to reach to the cells beneath and connect to them^53^.

In the current study, the haustoria of *C. planiflora* successfully penetrated stem epidermis and cortex and connected with the vascular bundles in all three cultivars (Helali, Giza 6, Gemmiza 1). However, Gemmiza 1 exhibited the most notable distortion of vascular bundles, with increased xylem-to-xylem connections established between host and dodder. In contrast, the Helali cultivar, although penetrated by haustoria, showed less distortion and more compact vascular bundles with fewer host-parasite xylary connections. This suggests that Helali may be more tolerant to dodder, as indicated by El-Refaey et al.^11^, who found that haustoria of dodder developed only through epidermis and cortex of the Helali cultivar stem without connecting to the vascular cylinder.

Hypertrophy of stem cortical cells was noticed in response to haustorial penetration, with the Helali cultivar showing the greatest increase in cortical layer thickness, while Gemmiza 1 exhibited the least. Such increase in cell size was associated with an overall increase in stem wall thickness. Many authors reported such hypertrophy in response to dodder penetration. For example, Farah and Ibrahim^52^ observed cortical cell enlargement in potato and tomato stems when penetrated with haustoria of *Cuscuta*. Likewise, Furuhashi et al.^56^ noted enlargement of cortical parenchyma in *Cuscuta*-infested stem of *Momordica charantia*. Moreover, elongation of cortical cells was noticed in stems of several species infested with *C. reflexa*^57^. Hypertrophy of cortex, observed in the Helali cultivar stem, is regarded as a plant defense mechanism against dodder invasion, potentially leading to distorted haustorial structures and disrupted connections with host vascular elements^52^. Additionally, increased lignification of xylem vessels was noticed in the stems of infested cultivars, especially Helali. This serves as another defense strategy via establishing a mechanical barrier against parasitic invasion and enhancing the rigidity and water capacity of xylem vessels^11,20,53,54,58^.

#### Role of *Fusarium* bioherbicide in enhancing stem anatomy under dodder infestation

Scarce literature is available on the effect of *Fusarium* spp. in controlling *Cuscuta* spp*.*, especially on the anatomy of host. Moreover, no reports investigated the Egyptian clover stem anatomy in response to *Fusarium* treatment as a dodder control method. However, they were reported to inhibit the growth of several weed species in pre-emergence stages^22,24^. Moreover, *Fusarium* is host-specific, which means that it could affect only the targeted weed species without affecting the host plant^22,27,46^.

Anatomically, Ndambi et al.^59^ studied the mode of action of *F. oxysporum* against *Striga hermonthica* in sorghum, finding that the fungal strain (Foxy 2) effectively invaded and digested xylem vessels in young seedlings, thereby reducing *Striga* infestation. Moreover, if *Striga* seeds germinated and infested sorghum, fungal hyphae were capable of clogging xylem vessels of *Striga,* causing its wilt and death, without affecting the sorghum plants^22,58,59^. In the current study, the stem anatomical structure of the three studied cultivars infested with *C. planiflora* and treated with *F. incarnatum*-based bioherbicide resembled that of control plants; free from parasitic weed and fungal invasions. This finding suggests that *F. incarnatum* effectively controls the parasitic plant during pre-emergence stages, highlighting its potential as a sustainable method for managing dodder infestations in Egyptian clover.

While *F. incarnatum* is not as widely studied as other *Fusarium* species for weed control, some literature indicates its potential as a biocontrol agent. Shabana et al.^60–64^ has proven successful use of *F. incarnatum* as a formulated granular herbicide for combating *Orobanche crenata*, a serious parasitic weed on leguminous plants. There is an increasing interest in exploring lesser-known fungal species, including *F. incarnatum*, for their potential in integrated weed management systems.

### Electrophoretic protein pattern

The electrophoretic analysis of protein patterns revealed distinct responses among the Egyptian clover cultivars to dodder infestation and bioherbicide treatment. The Helali cultivar maintained intact protein bands in all cases (control, infested, infested-bioherbicide treated). In contrast, the infested Gemmiza 1 cultivar exhibited the absence of two protein bands compared to its control, however, the infested-treated Gemmiza 1 was missing only one band. The unchanged protein expression pattern in the Helali cultivar suggests a higher tolerance to dodder infestation, supported by literature indicating elevated levels of phenolic compounds in that cultivar^11^. Moreover, the Helali cultivar is genetically diverse compared to Giza 6 and Gemmiza 1^10^. However, molecular and biochemical explorations are still deficient on this side in relevance with dodder infestation.

On the biochemical level, phenolic derivatives contribute to plant defense mechanisms via allelopathy^11,38^. Moreover, changes in phenolic composition in host exudates can disrupt host-parasite signaling, hindering infection^27^, which may explain the tolerance of the Helali cultivar to dodder infestation. Supporting literature includes Kaiser et al.^54^, who reported increased secretion of soluble phenylpropanoids in tomato infested with *C. reflexa*. This is linked to enhanced peroxidase activity that reinforces cell walls against parasite penetration via sealing of infection sites. Zhou et al.^38^ also noted that enhanced phenylpropanoid biosynthesis and increased transcription of lignin-biosynthesis-related genes serve as defense mechanisms against dodder infestation in white clover.

During infestation, *Cuscuta* haustorium penetration up-regulates genes encoding enzymes that loosen host cell walls, allowing penetration^65^. Moreover, connections established between host and *Cuscuta* may allow the transfer of proteins, where microRNAs potentially travel from *Cuscuta* to host, knocking out their defense-related genes^66^. This could explain the absence of some bands in case of infestation in Gemmiza 1 and Giza 6 cultivars.

*Fusarium* spp. are considered significant bio-agents used in the control of parasitic weeds, exerting direct and indirect effects on weed species^22,27^. Direct mechanisms involve pathogenicity to weed species either by suppression of germination and/or its inhibition at various life stages. For example, the Nep1 protein isolated from *F. oxysporum* liquid extract has been shown to induce necrosis in leaves of many weed species by stimulating ethylene biosynthesis^67–69^. Additionally, *F. oxysporum* is host specific, inhibiting growth of specific weeds without harming host or non-target plants. For example, *F. oxysporum* f.sp. *strigae* inhibits *Striga* spp. by inducing an overproduction of certain amino acids such as tyrosine, leucine and methionine, creating a metabolic imbalance in the weed^27^.

The secretion of secondary metabolites by *Fusarium* species is another direct mode of action by inhibiting germination and disrupting host-parasite interactions^70^. Changes in the chemical composition of plant exudates can drastically affect the germination and the development of weed haustoria, suppressing its growth and attachment to host plants^27^.

Indirect mechanisms of *Fusarium* include enhancing host resistance and defense system. In that concern, Ji et al.^71^ created a model patho-system using a pathogenicity factor of *F. oxysporum* to investigate the molecular mechanisms of pathogen-host interactions. The authors suggested that effector proteins produced by pathogens to suppress host innate immunity could be transferred from the parasite to the host plant, impairing the host’s immune response. This could potentially explain the changes in the protein pattern of Gemmiza 1 and Giza 6 cultivars in response to *Fusarium*-bioherbicide treatment.

The results of the current study provide a valuable foundation for future investigations into the molecular interactions between *Cuscuta* and Egyptian clover, as well as the potential role of *F. incarnatum* as a bioherbicide in suppressing *Cuscuta* germination and growth.

### Economic evaluation of Si-NPs and *Fusarium* spp. as potential herbicides

From a practical standpoint, the use of Si-NPs and *Fusarium* spp. as herbicides offers promising scalability for field application. Unlike synthetic herbicides, which incur recurring costs and pose environmental and health risks, Si-NPs and *Fusarium*-based controls can be cost-effective owing to their relatively low production costs^72^. Moreover, these treatments require fewer applications compared to chemical herbicide, which often necessitates multiple applications per season to acheive the desired effects. Furthermore, both treatments are highly competitive with chemical herbicides in terms of production costs and net income. In the current study, the application of Si-NPs as a herbicide resulted in the highest clover biomass and net income compared to other treatments. *Fusarium* bioherbicide could also be comparable with both Si-NPs and glyphosate treatments with lower yield production and net income, while exhibiting the lowest production cost.

In the long run, Si-NPs and *Fusarium* bioherbicides could potentially contribute to environmental sustainability and reduction of crop production costs. For example, Si-NPs have been shown to improve soil structure by enhancing aggregate stability and increasing nutrient availability. This can lead to better water retention and aeration, which are vital for healthy soil ecosystems^73^. Long-term application of Si-NPs may contribute to sustained soil fertility by promoting beneficial microbial activity and enhancing organic matter decomposition^73^. Studies indicate that Si-NPs can enhance the abundance of beneficial microbes while suppressing pathogenic species, thereby promoting a healthier soil microbiome^73^. Conversely, the application of *Fusarium* may selectively affect certain microbial populations, potentially altering community dynamics^74^. The functional diversity of soil microbes is crucial for ecosystem services such as nutrient cycling and organic matter decomposition^73^. The host specificity of *Fusarium* spp. could minimize adverse effects on non-target species and beneficial soil microbes, thereby supporting sustainable agricultural practices^75^. However, the impact of *Fusarium* on microbial functionality, particularly regarding its role as a biocontrol agent, warrants careful evaluation to avoid unintended consequences on soil health^74^.

In conclusion, further field-scale studies and cost–benefit analyses are recommended to fully establish the economic feasibility of Si-NPs and *Fusarium* herbicides and their farmer adoption potential.

## Conclusion

Dodder is the major parasitic plant species affecting the productivity of Egyptian clover, a major forage crop in Egypt. The current investigation highlighted significant reductions in biomass and seed yield of clover plants infested with dodder compared to non-infested ones. On the other hand, chemical, biological and nano-control treatments effectively managed dodder infestations and minimized yield losses. The selection of tolerant cultivars is also critical for effective control. The Helali cultivar demonstrated the highest tolerance to dodder, as witnessed by reduced dodder growth and enhanced clover productivity. Results of anatomical investigations and electrophoretic protein patterns further support the increased tolerance of Helali compared to other cultivars. Among the control methods, the application of Si-NPs at 30 g fed^−1^ was the most effective in reducing dodder mass, matching the effects of glyphosate. Notably, Si-NPs at 20 and 30 g fed^−1^ scored the highest mean values for fresh, dry, and seed yields of clover under infestation conditions, particularly in interactions with Helali and Gemmiza 1 cultivars. The biological control using *F. incarnatum* as a granular bioherbicide at rates of 20 and 30 kg fed^−1^ showed significant efficacy in reducing dodder biomass and enhancing clover productivity. The anatomical investigations and electrophoretic protein pattern provided further evidence of the potential effectiveness of *F. incarnatum* as a bioherbicide in dodder control. In conclusion, the Helali cultivar could be recommended as a tolerant option for dodder infestation. An integrated control program utilizing both nano- and bio-treatments presents a cost effective and environmentally safe alternative to chemical herbicides for managing dodder and other weeds infestations.

## Supplementary Information

Below is the link to the electronic supplementary material.

Supplementary Material 1

## Data Availability

Data could be available from the corresponding author on reasonable request.
