# Supplementary material for: Controlling dodder (Cuscuta planiflora) in Egyptian clover with silica nanoparticles and a novel bioherbicide
Source: Sci Rep. 2025 Aug 25;15:31223. doi: 10.1038/s41598-025-16004-6 (PMC12379218; doi:10.1038/s41598-025-16004-6)
Supplement: Supplementary file 1 — Supplementary Material 1 [file 41598_2025_16004_MOESM1_ESM.docx]

**Supplementary Table 1** Effect of the interaction between Egyptian clover cultivars and dodder control treatments on fresh and dry weights on dodder in the 2021/22 and 2022/23 seasons

| **Cultivars** | **Treatments** | **Rate fed^-1^** | **Fresh and dry weights of dodder weed (g m^-2^)** | | | | | | | |
| --- | --- | --- | --- | --- | --- | --- | --- | --- | --- | --- |
|  |  |  | **First cut** | | **Second cut** | | **Third cut** | | **Fourth cut** | |
|  |  |  | **FW** | **DW** | **FW** | **DW** | **FW** | **DW** | **FW** | **DW** |
|  |  |  | **2021/22 Season** | | | | | | | |
| **Gemmiza 1** | **Bioherbicide** | **10 kg** | 52.3^d^ | 11.5^cd^ | 106.3^e^ | 20.0^c^ | 376.3^c^ | 73.7^cd^ | 546.3^e^ | 95.1^cd^ |
|  | **Bioherbicide** | **20 kg** | 42.8^ef^ | 10.3^cde^ | 92.7^f^ | 16. 8^cde^ | 362.7^de^ | 70.5^cde^ | 532.7^f^ | 91.9^cde^ |
|  | **Bioherbicide** | **30 kg** | 37.3^fg^ | 7.8^f^ | 79.2^i^ | 15.1^cdefg^ | 350.8^fg^ | 68.2^de^ | 520.8^h^ | 89.6^de^ |
|  | **Si-NPs** | **14 g** | 20.0^i^ | 3.7^ghi^ | 55.3^kl^ | 10.6^fghij^ | 116.9^ijk^ | 37.1^f^ | 161.9^klm^ | 34.8^f^ |
|  | **Si-NPs** | **22 g** | 16.1^ijk^ | 2.2^hij^ | 51.7^mn^ | 9.5^ghij^ | 113.4^jkl^ | 35.9^f^ | 158.4^mn^ | 33.6^f^ |
|  | **Si-NPs** | **30 g** | 11.6^kl^ | 1.6^ij^ | 48.6^n^ | 9.1^hij^ | 110.3^kl^ | 35.5^f^ | 155.3^n^ | 33.2^f^ |
|  | **Glyphosate** | **33.1 g** | 17.2^ij^ | 1.8^ij^ | 52.3^lm^ | 10.1^ghij^ | 114.0^ijk^ | 36. 5^f^ | 159.0^m^ | 34.2^f^ |
|  | **Infested** |  | 120.6^a^ | 41.7^a^ | 355.3^b^ | 75.1^a^ | 568.7^a^ | 121.5^a^ | 688.7^b^ | 137.6^a^ |
|  | **Non-infested** |  | 0.0^m^ | 0.0^j^ | 0.0^q^ | 0.0^k^ | 0.0^o^ | 0.0^g^ | 0.0^q^ | 0.0^g^ |
| **Helali** | **Bioherbicide** | **10 kg** | 41.5^ef^ | 9.7^def^ | 88.2^g^ | 16.2^cdef^ | 358.2^def^ | 69.6^cd^ | 528.2^g^ | 91.0^c^ |
|  | **Bioherbicide** | **20 kg** | 34.9^g^ | 8.4^ef^ | 79.4^i^ | 14.0^defg^ | 349.4^g^ | 67.4^e^ | 519.4^h^ | 88.8^e^ |
|  | **Bioherbicide** | **30 kg** | 28.2^h^ | 4.8^g^ | 63.4^j^ | 11.9^defghi^ | 335.1^h^ | 65.3^e^ | 505.1^i^ | 86.7^e^ |
|  | **Si-NPs** | **14 g** | 18.3^ij^ | 2.7^ghi^ | 43.3^o^ | 8.1^hij^ | 105.0^lm^ | 34.5^f^ | 150.0^o^ | 32.2^f^ |
|  | **Si-NPs** | **22 g** | 14.1^jk^ | 1.7^ij^ | 35.6^p^ | 6.3^ij^ | 97.2^mn^ | 32.5^f^ | 142.2^p^ | 30.4^f^ |
|  | **Si-NPs** | **30 g** | 9.5^l^ | 1.4^ij^ | 33.2^p^ | 5.8^jk^ | 94.9^n^ | 32.2^f^ | 139.9^p^ | 29.9^f^ |
|  | **Glyphosate** | **33.1 g** | 14.7^ijkl^ | 1.7^hij^ | 35.5^p^ | 6.4^ij^ | 97.1^mn^ | 32.6^f^ | 142.1^p^ | 30.5^f^ |
|  | **Infested** |  | 99.2^b^ | 37.7^b^ | 313.2^c^ | 65.2^b^ | 526.5^b^ | 111.6^b^ | 646.5^c^ | 127. 7^b^ |
|  | **Non-infested** |  | 0.0^m^ | 0.0^j^ | 0.0^q^ | 0.0^k^ | 0.0^o^ | 0.0^g^ | 0.0^q^ | 0.0^g^ |
| **Giza 6** | **Bioherbicide** | **10 kg** | 61.8^c^ | 12.2^c^ | 111.5^d^ | 20.9^c^ | 381.5^c^ | 74.3^c^ | 551.5^d^ | 95.7^c^ |
|  | **Bioherbicide** | **20 kg** | 56.6^cd^ | 10.3^cde^ | 96.0^f^ | 17.4^cd^ | 366.0^d^ | 70.8^cde^ | 536.0^f^ | 92.2^cde^ |
|  | **Bioherbicide** | **30 kg** | 45.1^e^ | 8.5^ef^ | 84.1^h^ | 16.1^cdef^ | 355.8^efg^ | 69.5^cde^ | 525.8^g^ | 90.9^cde^ |
|  | **Si-NPs** | **14 g** | 25.7^h^ | 4.3^gh^ | 60.9^j^ | 11.6^defghij^ | 122.5^i^ | 38.0^f^ | 167.5^j^ | 35.7^f^ |
|  | **Si-NPs** | **22 g** | 20.0^i^ | 3.3^ghi^ | 56.9^k^ | 10.4^fghij^ | 118.5^ijk^ | 36.8^f^ | 163.5^kl^ | 34.5^f^ |
|  | **Si-NPs** | **30 g** | 14.4^jkl^ | 2.0^ij^ | 53.4^lm^ | 10.0^ghij^ | 115.1^ijk^ | 36.4^f^ | 160.1^lm^ | 34.1^f^ |
|  | **Glyphosate** | **33.1 g** | 18.3^ij^ | 2.2^hij^ | 57.4^k^ | 11.0^efghij^ | 119.1^ij^ | 37.4^f^ | 164.1^jk^ | 35.1^f^ |
|  | **Infested** |  | 123.8^a^ | 43.3^a^ | 361.1^a^ | 78.0^a^ | 574.4^a^ | 124.4^a^ | 694.4^a^ | 140.5^a^ |
|  | **Non-infested** |  | 0.0^m^ | 0.0^j^ | 0.0^q^ | 0.0^k^ | 0.0^o^ | 0.0^g^ | 0.0^q^ | 0.0^g^ |
|  | **LSD _0.05_** |  | **5.52** | **2.39** | **3.42** | **5.97** | **8.53** | **6.00** | **3.52** | **5.91** |
|  |  |  | **2022/23 Season** | | | | | | | |
| **Gemmiza 1** | **Bioherbicide** | **10 kg** | 63.9^d^ | 14.8^cd^ | 121.7^d^ | 22.83^d^ | 430.6^d^ | 68.41^d^ | 625.3^d^ | 108.71^d^ |
|  | **Bioherbicide** | **20 kg** | 52.2^ef^ | 13.4^cde^ | 106.2^ef^ | 19.22^ef^ | 415.3^ef^ | 64.80^e^ | 609.9^ef^ | 105.10^e^ |
|  | **Bioherbicide** | **30 kg** | 45.6^fg^ | 10.3^e^ | 90.4^h^ | 17.29^fg^ | 401.2^h^ | 62.13^fg^ | 595.9^hi^ | 102.42^f^ |
|  | **Si-NPs** | **14 g** | 24.3^jk^ | 4.8^fgh^ | 63.3^jk^ | 12.07^hi^ | 134.0^jk^ | 26.62^ijk^ | 185.3^lmn^ | 39.83^hij^ |
|  | **Si-NPs** | **22 g** | 19.7^klm^ | 2.9^ghi^ | 59.2^kl^ | 10.84^ij^ | 129.9^kl^ | 25.19^jkl^ | 181.2^mn^ | 38.39^hij^ |
|  | **Si-NPs** | **30 g** | 14.2^lm^ | 2.1^hi^ | 55.6^lm^ | 10.44^ij^ | 126.3^lm^ | 24.78^kl^ | 177.6^no^ | 37.99^ij^ |
|  | **Glyphosate** | **33.1 g** | 21.0^kl^ | 2.3^hi^ | 59.7^kl^ | 11.46^hij^ | 130.4^kl^ | 25.81^ijk^ | 181.7^lmn^ | 39.01^hij^ |
|  | **Infested** |  | 147.6^a^ | 51.0^a^ | 400.4^b^ | 86.02^b^ | 553.9^c^ | 123.24^b^ | 783.7^b^ | 157.48^b^ |
|  | **Non-infested** |  | 0.0^n^ | 0.0^i^ | 0.0^o^ | 0.0^m^ | 0.0^o^ | 0.0^n^ | 0.0^q^ | 0.0^l^ |
| **Helali** | **Bioherbicide** | **10 kg** | 50.6^efg^ | 12.7^de^ | 100.8^fg^ | 18.46^ef^ | 409.8^fg^ | 63.68^ef^ | 604.4^fg^ | 103.99^ef^ |
|  | **Bioherbicide** | **20 kg** | 42.6^gh^ | 11.0^e^ | 90.6^h^ | 15.98^g^ | 399.7^h^ | 61.20^g^ | 594.3^i^ | 101.50^fg^ |
|  | **Bioherbicide** | **30 kg** | 34.4^hi^ | 6.4^f^ | 72.6^i^ | 13.56^h^ | 383.3^i^ | 58.78^h^ | 578.0^j^ | 99.08^g^ |
|  | **Si-NPs** | **14 g** | 22.4^kl^ | 3.5^fgh^ | 49.6^m^ | 9.24^jk^ | 120.4^m^ | 23.59^l^ | 171.6^o^ | 36.79^jk^ |
|  | **Si-NPs** | **22 g** | 17.2^klm^ | 2.3^hi^ | 40.7^n^ | 7.26^kl^ | 111.4^n^ | 21.61^m^ | 162.7^p^ | 34.81^k^ |
|  | **Si-NPs** | **30 g** | 11.6^m^ | 1.9^hi^ | 38.1^n^ | 6.65^l^ | 108.8^n^ | 21.00^m^ | 160.1^p^ | 34.21^k^ |
|  | **Glyphosate** | **33.1 g** | 17.9^klm^ | 2.3^hi^ | 40.6^n^ | 7.28^kl^ | 111.4^n^ | 21.63^m^ | 162.6^p^ | 34.83^k^ |
|  | **Infested** |  | 121.4^b^ | 46.0^b^ | 354.9^c^ | 74.67^c^ | 605.6^b^ | 111.89^c^ | 732.1^c^ | 146.13^c^ |
|  | **Non-infested** |  | 0.0^n^ | 0.0^i^ | 0.0^o^ | 0.0^m^ | 0.0^o^ | 0.0^n^ | 0.0^q^ | 0.0^l^ |
| **Giza 6** | **Bioherbicide** | **10 kg** | 75.4^c^ | 16.0^c^ | 127.6^d^ | 23.91^d^ | 436.5^d^ | 69.13^d^ | 631.2^d^ | 109.43^d^ |
|  | **Bioherbicide** | **20 kg** | 69.1^cd^ | 13.5^cde^ | 109.9^e^ | 19.91^e^ | 419.0^e^ | 65.12^e^ | 613.6^e^ | 105.43^e^ |
|  | **Bioherbicide** | **30 kg** | 55.0^e^ | 11.2^e^ | 96.1^gh^ | 18.36^ef^ | 406.9^gh^ | 63.58^ef^ | 601.6^gh^ | 103.88^ef^ |
|  | **Si-NPs** | **14 g** | 31.4^ij^ | 5.7^fg^ | 69.7^ij^ | 13.31^h^ | 140.4^j^ | 27.66^i^ | 191.7^k^ | 40.87^h^ |
|  | **Si-NPs** | **22 g** | 24.4^jk^ | 4.4^fgh^ | 65.1^jk^ | 11.91^hi^ | 135.8^jk^ | 26.26^ijk^ | 187.1^kl^ | 39.46^hij^ |
|  | **Si-NPs** | **30 g** | 17.5^klm^ | 2.7^ghi^ | 61.1^kl^ | 11.45^hij^ | 131.9^kl^ | 25.80^ijk^ | 183.1^lmn^ | 39.01^hij^ |
|  | **Glyphosate** | **33.1 g** | 22.4^kl^ | 2.9^ghi^ | 65.6^jk^ | 12.55^hi^ | 136.3^jk^ | 26.90^ij^ | 187.6^kl^ | 40.11^hi^ |
|  | **Infested** |  | 151.8^a^ | 53.0^a^ | 415.2^a^ | 89.34^m^ | 661.0^a^ | 126.56^a^ | 790.4^a^ | 160.80^a^ |
|  | **Non-infested** |  | 0.0^n^ | 0.0^i^ | 0.0^o^ | 0.0^l^ | 0.0^o^ | 0.0^n^ | 0.0^q^ | 0.0^l^ |
|  | **LSD _0.05_** |  | **8.23** | **3.25** | **6.82** | **2.33** | **7.25** | **1.90** | **6.04** | **2.81** |

FW= Fresh weight, DW= Dry weight. Values (means) followed by different letter(s) are significantly different according to LSD at p ≤ 0.05.

**Supplementary Table 2** Effect of the interaction between Egyptian clover cultivars and dodder control treatments on fresh and dry weights of clover in the 2021/22 and 2022/23 seasons

| **Cultivars** | **Treatments** | **Rate fed^-1^** | **Fresh and dry weights of clover (ton fed^-1^)** | | | | | | | |
| --- | --- | --- | --- | --- | --- | --- | --- | --- | --- | --- |
|  |  |  | **First cut** | | **Second cut** | | **Third cut** | | **Fourth cut** | |
|  |  |  | **FW** | **DW** | **FW** | **DW** | **FW** | **DW** | **FW** | **DW** |
|  |  |  | **2021/22 Season** | | | | | | | |
| **Gemmiza 1** | **Bioherbicide** | **10 kg** | 6.37^jk^ | 1.13^k^ | 9.86^j^ | 1.63^l^ | 8.89^i^ | 1.50^jk^ | 8.29^k^ | 1.37^ef^ |
|  | **Bioherbicide** | **20 kg** | 6.89^hi^ | 1.19^j^ | 10.52^hi^ | 1.70^kl^ | 9.49^h^ | 1.56^ij^ | 8.86^j^ | 1.43^de^ |
|  | **Bioherbicide** | **30 kg** | 6.94^ghi^ | 1.19^j^ | 10.68^gh^ | 1.73^jk^ | 9.63^h^ | 1.59^i^ | 8.99^j^ | 1.45^de^ |
|  | **Si-NPs** | **14 g** | 7.56^def^ | 1.31^cde^ | 11.64^f^ | 1.86^efgh^ | 12.21^ef^ | 1.92^fg^ | 12.36^gh^ | 1.96^c^ |
|  | **Si-NPs** | **22 g** | 7.70^cde^ | 1.33^c^ | 12.16^de^ | 1.92^cde^ | 12.74^d^ | 1.99^cde^ | 12.92^e^ | 2.03^bc^ |
|  | **Si-NPs** | **30 g** | 7.70^cde^ | 1.32^cd^ | 12.20^cde^ | 1.93^bcde^ | 12.76^cd^ | 2.00^cd^ | 12.97^e^ | 2.04^bc^ |
|  | **Glyphosate** | **33.1 g** | 7.69^cde^ | 1.33^c^ | 12.17^de^ | 1.93^bcde^ | 12.71^d^ | 2.00^cd^ | 12.92^e^ | 2.04^bc^ |
|  | **Infested** |  | 5.57^l^ | 1.00^m^ | 7.52^l^ | 1.37^n^ | 7.45^k^ | 1.34^l^ | 7.41^l^ | 1.27^fg^ |
|  | **Non-infested** |  | 8.39^ab^ | 1.37^b^ | 12.62^b^ | 1.97^abcd^ | 13.28^b^ | 2.05^bc^ | 13.81^b^ | 2.11^ab^ |
| **Helali** | **Bioherbicide** | **10 kg** | 6.70^ij^ | 1.20^j^ | 10.26^i^ | 1.73^jk^ | 9.42^h^ | 1.59^i^ | 8.91^j^ | 1.45^de^ |
|  | **Bioherbicide** | **20 kg** | 7.19^fgh^ | 1.25^i^ | 11.04^g^ | 1.81^hij^ | 10.16^g^ | 1.67^h^ | 9.56^i^ | 1.52^d^ |
|  | **Bioherbicide** | **30 kg** | 7.23^fgh^ | 1.26^hi^ | 11.12^g^ | 1.83^fgh^ | 10.26^g^ | 1.68^h^ | 9.62^i^ | 1.54^d^ |
|  | **Si-NPs** | **14 g** | 8.10^bc^ | 1.37^b^ | 12.45^bcd^ | 2.00^abc^ | 13.02^bc^ | 2.07^ab^ | 13.47^cd^ | 2.11^ab^ |
|  | **Si-NPs** | **22 g** | 7.95^bcd^ | 1.39^b^ | 12.51^bc^ | 2.01^ab^ | 13.10^b^ | 2.08^ab^ | 13.54^cd^ | 2.12^ab^ |
|  | **Si-NPs** | **30 g** | 8.02^bc^ | 1.39^b^ | 12.56^bc^ | 2.00^abc^ | 13.10^b^ | 2.07^ab^ | 13.60^bc^ | 2.11^ab^ |
|  | **Glyphosate** | **33.1 g** | 7.97^bcd^ | 1.39^b^ | 12.50^bcd^ | 2.00^abc^ | 13.10^b^ | 2.07^ab^ | 13.53^cd^ | 2.11^ab^ |
|  | **Infested** |  | 6.04^k^ | 1.05^l^ | 8.32^k^ | 1.51^m^ | 8.27^j^ | 1.46^k^ | 8.13^k^ | 1.37^ef^ |
|  | **Non-infested** |  | 8.70^a^ | 1.43^a^ | 13.04^a^ | 2.05^a^ | 13.61^a^ | 2.13^a^ | 14.33^a^ | 2.20^a^ |
| **Giza 6** | **Bioherbicide** | **10 kg** | 6.15^k^ | 1.11^k^ | 9.77^j^ | 1.64^l^ | 8.85^i^ | 1.50^jk^ | 8.22^k^ | 1.38^ef^ |
|  | **Bioherbicide** | **20 kg** | 6.26^jk^ | 1.12^k^ | 10.36^hi^ | 1.74^ijk^ | 9.39^h^ | 1.58^i^ | 8.77^j^ | 1.46^de^ |
|  | **Bioherbicide** | **30 kg** | 6.34^jk^ | 1.13^k^ | 10.50^hi^ | 1.74^ijk^ | 9.60^h^ | 1.59^i^ | 8.83^j^ | 1.46^de^ |
|  | **Si-NPs** | **14 g** | 7.13^fghi^ | 1.27^ghi^ | 11.60^f^ | 1.88^efgh^ | 12.10^f^ | 1.93^efg^ | 12.32^h^ | 1.99^c^ |
|  | **Si-NPs** | **22 g** | 7.27^efgh^ | 1.28^fgh^ | 11.83^ef^ | 1.91^def^ | 12.47^de^ | 1.96^def^ | 12.56^fg^ | 2.02^bc^ |
|  | **Si-NPs** | **30 g** | 7.36^efg^ | 1.29^efg^ | 11.87^ef^ | 1.90^defg^ | 12.47^de^ | 1.96^def^ | 12.68^f^ | 2.01^bc^ |
|  | **Glyphosate** | **33.1 g** | 7.31^efgh^ | 1.28^fgh^ | 11.82^ef^ | 1.90^defg^ | 12.41^e^ | 1.96^def^ | 12.54^fgh^ | 2.01^bc^ |
|  | **Infested** |  | 5.33^l^ | 0.97^n^ | 7.43^l^ | 1.64^l^ | 7.32^k^ | 1.31^l^ | 7.18^l^ | 1.25^g^ |
|  | **Non-infested** |  | 8.24^b^ | 1.30^def^ | 12.39^bcd^ | 1.82^ghi^ | 13.04^bc^ | 1.89^g^ | 13.35^d^ | 1.95^c^ |
|  | **LSD 0.05** |  | **0.45** | **0.02** | **0.38** | **0.084** | **0.29** | **0.065** | **0.235** | **0.11** |
|  |  |  | **2022/23 Season** | | | | | | | |
| **Gemmiza 1** | **Bioherbicide** | **10 kg** | 5.41^ij^ | 0.99^j^ | 8.67^lm^ | 1.43^j^ | 7.83^l^ | 1.32^j^ | 7.30^j^ | 1.20^fg^ |
|  | **Bioherbicide** | **20 kg** | 5.85^gh^ | 1.04^i^ | 9.26^jk^ | 1.50^hij^ | 8.35^k^ | 1.38^ij^ | 7.80^i^ | 1.26^ef^ |
|  | **Bioherbicide** | **30 kg** | 5.90^gh^ | 1.05^i^ | 9.40^ijk^ | 1.52^ghij^ | 8.47^k^ | 1.40^i^ | 7.91^i^ | 1.28^ef^ |
|  | **Si-NPs** | **14 g** | 6.43^cdef^ | 1.15^def^ | 10.24^efg^ | 1.63^def^ | 10.74^hi^ | 1.69^fg^ | 10.88^g^ | 1.73^d^ |
|  | **Si-NPs** | **22 g** | 6.55^bcd^ | 1.17^cd^ | 10.70^bcdef^ | 1.69^bcde^ | 11.21^def^ | 1.75^def^ | 11.37^ef^ | 1.79^bcd^ |
|  | **Si-NPs** | **30 g** | 6.54^bcde^ | 1.16^de^ | 10.74^bcde^ | 1.70^bcd^ | 11.23^cdef^ | 1.76^cde^ | 11.41^de^ | 1.80^bcd^ |
|  | **Glyphosate** | **33.1 g** | 6.53^bcde^ | 1.17^cd^ | 10.71^bcdef^ | 1.70^bcd^ | 11.19^efg^ | 1.75^def^ | 11.37^ef^ | 1.79^bcd^ |
|  | **Infested** |  | 4.57^kl^ | 0.85^kl^ | 6.39^o^ | 1.17^l^ | 6.33^n^ | 1.14^l^ | 6.30^l^ | 1.08^hi^ |
|  | **Non-infested** |  | 7.13^a^ | 1.21^bc^ | 11.11^ab^ | 1.73^abc^ | 11.69^b^ | 1.80^bcd^ | 12.15^b^ | 1.85^abc^ |
| **Helali** | **Bioherbicide** | **10 kg** | 5.70^hi^ | 1.06^hi^ | 9.03^klm^ | 1.52^ghij^ | 8.29^k^ | 1.40^i^ | 7.84^i^ | 1.28^ef^ |
|  | **Bioherbicide** | **20 kg** | 6.11^efgh^ | 1.10^gh^ | 9.71^hij^ | 1.59^fgh^ | 8.94^j^ | 1.47^h^ | 8.41^h^ | 1.34^e^ |
|  | **Bioherbicide** | **30 kg** | 6.15^defg^ | 1.11^gh^ | 9.79^ghi^ | 1.61^defg^ | 9.03^j^ | 1.48^h^ | 8.47^h^ | 1.35^e^ |
|  | **Si-NPs** | **14 g** | 6.88^b^ | 1.21^bc^ | 10.96^bc^ | 1.76^ab^ | 11.46^bcde^ | 1.82^abc^ | 11.86^bc^ | 1.85^ab^ |
|  | **Si-NPs** | **22 g** | 6.76^bc^ | 1.22^ab^ | 11.01^ab^ | 1.77^ab^ | 11.53^bc^ | 1.83^ab^ | 11.91^bc^ | 1.86^ab^ |
|  | **Si-NPs** | **30 g** | 6.82^bc^ | 1.23^ab^ | 11.05^ab^ | 1.76^ab^ | 11.53^bc^ | 1.82^abc^ | 11.97^bc^ | 1.86^ab^ |
|  | **Glyphosate** | **33.1 g** | 6.78^bc^ | 1.22^ab^ | 11.00^ab^ | 1.76^ab^ | 11.53^bc^ | 1.82^abc^ | 11.91^bc^ | 1.85^abc^ |
|  | **Infested** |  | 4.96^k^ | 0.89^k^ | 7.08^n^ | 1.28^k^ | 7.03^m^ | 1.24^k^ | 6.91^k^ | 1.16^gh^ |
|  | **Non-infested** |  | 7.40^a^ | 1.26^a^ | 11.48^a^ | 1.81^a^ | 11.98^a^ | 1.88^a^ | 12.61^a^ | 1.93^a^ |
| **Giza 6** | **Bioherbicide** | **10 kg** | 5.23^j^ | 0.97^j^ | 8.60^m^ | 1.45^ij^ | 7.79^l^ | 1.32^j^ | 7.23^j^ | 1.22^fg^ |
|  | **Bioherbicide** | **20 kg** | 5.32^ij^ | 0.99^j^ | 9.12^kl^ | 1.53^ghi^ | 8.27^k^ | 1.39^i^ | 7.71^i^ | 1.28^ef^ |
|  | **Bioherbicide** | **30 kg** | 5.39^ij^ | 0.99^j^ | 9.24^jk^ | 1.53^ghi^ | 8.45^k^ | 1.40^i^ | 7.77^i^ | 1.29^ef^ |
|  | **Si-NPs** | **14 g** | 6.07^fgh^ | 1.12^efg^ | 10.21^fgh^ | 1.66^cdef^ | 10.65^i^ | 1.70^efg^ | 10.84^g^ | 1.75^d^ |
|  | **Si-NPs** | **22 g** | 6.18^defg^ | 1.13^defg^ | 10.41^def^ | 1.68^bcdef^ | 10.97^fgh^ | 1.73^efg^ | 11.05^fg^ | 1.77^bcd^ |
|  | **Si-NPs** | **30 g** | 6.26^defg^ | 1.14^defg^ | 10.45^cdef^ | 1.68^bcdef^ | 10.98^fg^ | 1.73^efg^ | 11.16^efg^ | 1.76^cd^ |
|  | **Glyphosate** | **33.1 g** | 6.21^defg^ | 1.13^defg^ | 10.40^def^ | 1.68^bcdef^ | 10.92^ghi^ | 1.73^efg^ | 11.03^g^ | 1.76^cd^ |
|  | **Infested** |  | 4.37^l^ | 0.83^l^ | 6.32^o^ | 1.21^kl^ | 6.22^n^ | 1.11^l^ | 6.11^l^ | 1.06^i^ |
|  | **Non-infested** |  | 7.00^a^ | 1.15^def^ | 10.86^bcd^ | 1.60^efg^ | 11.48^bcd^ | 1.67^g^ | 11.74^cd^ | 1.72^d^ |
|  | **LSD 0.05** |  | **0.43** | **0.04** | **0.51** | **0.091** | **0.27** | **0.063** | **0.333** | **0.098** |

FW= Fresh weight, DW= Dry weight. Values (means) followed by different letter(s) are significantly different according to LSD at p ≤ 0.05.

**Supplementary Table 3** Effect of the interaction between Egyptian clover cultivars and dodder control treatments on seed yield parameters of clover in the 2021/22 and 2022/23 seasons

| **Cultivars** | **Treatments** | **Rate fed^-1^** | **No seeds/head** | | **1000 Seed weight (g)** | | **Seed yield (kg fed^-1^)** | |
| --- | --- | --- | --- | --- | --- | --- | --- | --- |
|  |  |  | **1^st^ Season** | **2^nd^ Season** | **1^st^ Season** | **2^nd^ Season** | **1^st^ Season** | **2^nd^ Season** |
| **Gemmiza 1** | **Bioherbicide** | **10 kg** | 37.7^o^ | 39.6^no^ | 2.65^l^ | 2.77^k^ | 258.6^n^ | 271.6^j^ |
|  | **Bioherbicide** | **20 kg** | 45.7^jk^ | 47.9^ijk^ | 3.08^gh^ | 3.23^gh^ | 312.8^hi^ | 328.3^ef^ |
|  | **Bioherbicide** | **30 kg** | 39.3^n^ | 41.3^mn^ | 2.89^k^ | 3.03^j^ | 265.5^m^ | 279.0^ij^ |
|  | **Si-NPs** | **14 g** | 46.7^hij^ | 49.0^hij^ | 3.16^f^ | 3.33^ef^ | 319.4^fg^ | 335.2^e^ |
|  | **Si-NPs** | **22 g** | 50.3^d^ | 52.9^cde^ | 3.34^bc^ | 3.50^bc^ | 343.1^cd^ | 360.2^cd^ |
|  | **Si-NPs** | **30 g** | 48.7^efg^ | 51.1^efg^ | 3.27^de^ | 3.43^cd^ | 339.9^cd^ | 356.9^cd^ |
|  | **Glyphosate** | **33.1 g** | 49.3^def^ | 51.8^def^ | 3.30^cd^ | 3.47^bcd^ | 338.2^de^ | 355.0^cd^ |
|  | **Infested** |  | 34.7^p^ | 36.7^p^ | 2.37^op^ | 2.50^m^ | 253.7^n^ | 268.8^j^ |
|  | **Non-infested** |  | 53.3^b^ | 56.0^b^ | 3.42^a^ | 3.60^a^ | 357.0^b^ | 375.0^b^ |
| **Helali** | **Bioherbicide** | **10 kg** | 43.3^m^ | 45.5^l^ | 2.48^n^ | 2.60^l^ | 291.2^k^ | 305.9^h^ |
|  | **Bioherbicide** | **20 kg** | 49.7^de^ | 52.1^def^ | 2.97^j^ | 3.13^i^ | 333.6^e^ | 350.2^d^ |
|  | **Bioherbicide** | **30 kg** | 44.0^lm^ | 46.2^kl^ | 2.58^m^ | 2.73^k^ | 296.5^k^ | 311.4^gh^ |
|  | **Si-NPs** | **14 g** | 48.0^fgh^ | 50.4^fgh^ | 3.02^i^ | 3.20^hi^ | 322.0^f^ | 338.1^e^ |
|  | **Si-NPs** | **22 g** | 53.0^b^ | 55.6^b^ | 3.25^e^ | 3.43^cd^ | 341.7^cd^ | 358.7^cd^ |
|  | **Si-NPs** | **30 g** | 50.7^cd^ | 53.2^cd^ | 3.12^fg^ | 3.27^fgh^ | 341.3^cd^ | 358.5^cd^ |
|  | **Glyphosate** | **33.1 g** | 52.0^bc^ | 54.6^bc^ | 3.14^f^ | 3.30^fg^ | 344.6^c^ | 362.0^c^ |
|  | **Infested** |  | 40.3^n^ | 42.6^m^ | 2.34^mn^ | 2.47^m^ | 272.8^l^ | 288.1^i^ |
|  | **Non-infested** |  | 57.7^a^ | 60.6^a^ | 3.36^b^ | 3.53^ab^ | 368.9^a^ | 387.5^a^ |
| **Giza 6** | **Bioherbicide** | **10 kg** | 35.7^p^ | 37.4^p^ | 2.32^qr^ | 2.43^m^ | 239.6^op^ | 251.1^k^ |
|  | **Bioherbicide** | **20 kg** | 43.3^m^ | 45.5^l^ | 3.23^e^ | 3.40^de^ | 292.2^k^ | 306.7^h^ |
|  | **Bioherbicide** | **30 kg** | 36.0^p^ | 37.7^op^ | 2.39^o^ | 2.50^m^ | 241.3^o^ | 253.0^k^ |
|  | **Si-NPs** | **14 g** | 45.0^kl^ | 47.2^jkl^ | 2.87^k^ | 3.03^j^ | 305.6^j^ | 320.8^fg^ |
|  | **Si-NPs** | **22 g** | 46.0^ijk^ | 48.1^ijk^ | 3.16^f^ | 3.33^ef^ | 318.7^fgh^ | 333.6^e^ |
|  | **Si-NPs** | **30 g** | 45.7^jk^ | 47.9^ijk^ | 3.07^h^ | 3.20^hi^ | 307.4^ij^ | 322.1^fg^ |
|  | **Glyphosate** | **33.1 g** | 44.0^lm^ | 46.2^kl^ | 3.12^fg^ | 3.27^fgh^ | 314.4^gh^ | 330.2^ef^ |
|  | **Infested** |  | 34.7^p^ | 36.6^p^ | 2.29^r^ | 2.43^m^ | 234.3^p^ | 247.5^k^ |
|  | **Non-infested** |  | 47.3^ghi^ | 49.4^ghi^ | 3.27^de^ | 3.43^cd^ | 323.9^f^ | 338.4^e^ |
|  | **LSD _0.05_** |  | **1.44** | **2.01** | **0.04** | **0.09** | **5.94** | **11.08** |

1^st^ Season = 2021/22 season, 2^nd^ Season = 2022/ 23 season**.** Values (means) followed by different letter(s) are significantly different according to LSD at p ≤ 0.05.

**Supplementary Table 4** Histological measurements of stem (μm) for three cultivars of Egyptian clover (Helali, Giza 6, and Gemmiza 1) as healthy controls, dodder infested, and dodder infested-bioherbicide treated (20 kg fed^-1^). Values are means **±** standard error **(**S.E), n=3

| **Gemmiza 1 cultivar** | | | | | **Giza 6 cultivar** | | | | | **Helali cultivar** | | | | | **Histological parameters (μm)** |
| --- | --- | --- | --- | --- | --- | --- | --- | --- | --- | --- | --- | --- | --- | --- | --- |
| **% ± treat. to control** | **% ± infected to control** | **Bioherbicide treated**  **(20 kg fed^-1^)** | **Dodder infested** | **Control**  **(healthy)** | **% ± treat. to control** | **% ± infected to control** | **Bioherbicide treated**  **(20 kg fed^-1^)** | **Dodder infested** | **Healthy control** | **% ± treat. to control** | **% ± infected to control** | **Bioherbicide treated**  **(20 kg fed^-1^)** | **Dodder infested** | **Healthy control** |  |
| **- 11.5** | **- 16.6** | **3823.5±34.6** | **3603.5±64.9** | **4321.6±89.9** | **+ 1.1** | **- 0.1** | **3929.1±30.5** | **3881.0±39.3** | **3886.1±30.5** | **- 1.5** | **+ 25** | **4024.7±41.7** | **5105.9±37.0** | **4085.5±43.2** | **Stem diameter** |
| **+ 14.4** | **+ 68.7** | **725.2 ±21.4** | **1070.1±40.8** | **634.2 ±37.0** | **+ 18.1** | **+ 109.9** | **737.3 ±22.6** | **1310.2±27.6** | **624.1 ±11.0** | **- 1.6** | **+ 91.5** | **686.5 ±26.6** | **1336.1±29.9** | **697.6 ±24.6** | **Stem wall thickness** |
| **+ 8.1** | **+ 5.3** | **115.1 ±1.1** | **112.1 ±1.4** | **106.4 ±1.6** | **- 1.6** | **+ 65.0** | **113.8 ±2.1** | **180.2 ±5.2** | **109.2 ±1.1** | **- 1.6** | **+ 101.2** | **109.0 ±1.0** | **222.9 ±4.3** | **110.8 ±1.2** | **Cortex thickness** |

**Supplementary Table 5** Data analysis of the electrophoretic protein pattern by SDS-PAGE for total soluble protein of three Egyptian clover cultivars (Helali, Giza 6, and Gemmiza 1) as healthy controls, dodder infested, and dodder infested-bioherbicide treated (20 kg fed^-1^) , (+) sign indicates band presence, (-) sign indicates band absence

| **Molecular weight (KDa)** | **Helali (control)** | **Helali (infested)** | **Helali (treated)** | **Giza 6 (control)** | **Giza 6 (infested)** | **Giza 6 (treated)** | **Gemmiza 1 (control)** | **Gemmiza 1 (infested)** | **Gemmiza 1 (treated)** |
| --- | --- | --- | --- | --- | --- | --- | --- | --- | --- |
| **184.41** | **+** | **+** | **+** | **+** | **+** | **+** | **+** | **+** | **+** |
| **119.60** | **+** | **+** | **+** | **+** | **-** | **+** | **+** | **+** | **+** |
| **55.06** | **+** | **+** | **+** | **+** | **+** | **+** | **+** | **+** | **+** |
| **28.09** | **+** | **+** | **+** | **-** | **+** | **+** | **+** | **-** | **-** |
| **18.24** | **+** | **+** | **+** | **+** | **+** | **+** | **+** | **-** | **+** |
| **12.75** | **+** | **+** | **+** | **+** | **+** | **+** | **+** | **+** | **+** |
| **6.40** | **+** | **+** | **+** | **+** | **+** | **+** | **+** | **+** | **+** |
| **4.72** | **+** | **+** | **+** | **+** | **+** | **+** | **+** | **+** | **+** |
